# Identification of FLYWCH1 as a regulator of platinum-resistance in epithelial ovarian cancer

**DOI:** 10.1093/narcan/zcaf012

**Published:** 2025-04-04

**Authors:** Tabea L Fullstone, Helene Rohm, Till Kaltofen, Sophia Hierlmayer, Juliane Reichenbach, Simon Schweikert, Franziska Knodel, Ann-Kathrin Loeffler, Doris Mayr, Udo Jeschke, Sven Mahner, Mirjana Kessler, Fabian Trillsch, Philipp Rathert

**Affiliations:** Department of Molecular Biochemistry, Institute of Biochemistry, University of Stuttgart, 70569 Stuttgart, Germany; Department of Molecular Biochemistry, Institute of Biochemistry, University of Stuttgart, 70569 Stuttgart, Germany; Department of Obstetrics and Gynaecology, University Hospital, LMU Munich, 81377 Munich, Germany; Department of Surgery, University Hospital Regensburg, 93053 Regensburg, Germany; Department of Obstetrics and Gynaecology, University Hospital, LMU Munich, 81377 Munich, Germany; Department of Obstetrics and Gynaecology, University Hospital, LMU Munich, 81377 Munich, Germany; Department of Molecular Biochemistry, Institute of Biochemistry, University of Stuttgart, 70569 Stuttgart, Germany; Department of Molecular Biochemistry, Institute of Biochemistry, University of Stuttgart, 70569 Stuttgart, Germany; Department of Molecular Biochemistry, Institute of Biochemistry, University of Stuttgart, 70569 Stuttgart, Germany; Institute of Pathology, LMU Munich, 81377 Munich, Germany; Department of Obstetrics and Gynaecology, University Hospital, LMU Munich, 81377 Munich, Germany; Department of Obstetrics and Gynaecology, University Hospital Augsburg, 86156 Augsburg, Germany; Department of Obstetrics and Gynaecology, University Hospital, LMU Munich, 81377 Munich, Germany; Department of Obstetrics and Gynaecology, University Hospital, LMU Munich, 81377 Munich, Germany; Department of Obstetrics and Gynaecology, University Hospital, LMU Munich, 81377 Munich, Germany; Department of Molecular Biochemistry, Institute of Biochemistry, University of Stuttgart, 70569 Stuttgart, Germany

## Abstract

Platinum-based combination chemotherapy remains the backbone of first-line treatment for patients with advanced epithelial ovarian cancer (EOC). While most patients initially respond well to the treatment, patients with relapse ultimately develop platinum resistance. This study identified FLYWCH-type zinc finger-containing protein 1 (FLYWCH1) as an important regulator in the resistance development process. We showed that the loss of FLYWCH1 promotes platinum resistance in EOC cells, and the low FLYWCH1 expression is correlated with poor prognosis of EOC patients. In platinum-sensitive cells, FLYWCH1 colocalizes with H3K9me3, but this association is significantly reduced when cells acquire resistance. The suppression of FLYWCH1 induces gene expression changes resulting in the deregulation of pathways associated with resistance. In line with its connection to H3K9me3, FLYWCH1 induces gene silencing in a synthetic reporter assay and the suppression of FLYWCH1 alters H3K9me3 at promoter regions and repeat elements. The loss of FLYWCH1 leads to the derepression of LTR and Alu repeats, thereby increasing transcriptional plasticity and driving the resistance development process. Our data highlight the importance of FLYWCH1 in chromatin biology and acquisition of platinum resistance through transcriptional plasticity and propose FLYWCH1 as a potential biomarker for predicting treatment responses in EOC patients.

## Graphical Abstract

**Graphical Abstract:**
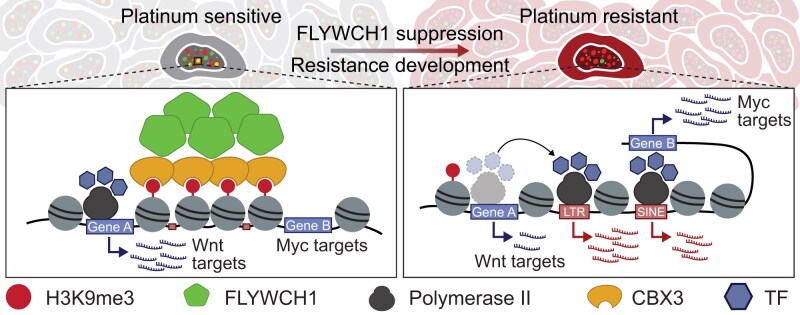

## Introduction

With 324 398 new cases and 206 839 deaths in 2022, epithelial ovarian cancer (EOC) is the third most common gynaecologic cancer in women, with the worst prognosis and highest mortality rate among gynaecologic malignancies [1, 2]. Around 70% of patients with ovarian cancer are diagnosed in advanced stages of the disease [3], with a corresponding poor prognosis. The standard of care for ovarian cancer consists of primary debulking surgery, followed by first-line platinum-based chemotherapy in combination with paclitaxel and bevacizumab [4]. This treatment can be extended by poly (ADP-ribose) polymerase (PARP)-inhibitor maintenance therapy depending on the Breast Cancer gene 1/2 (BRCA1/2) mutational and homologous recombination deficiency (HRD) status as well as the clinical profile of the patient [5]. Patients with recurrent disease are treated with other agents, such as gemcitabine or pegylated liposomal doxorubicin (PLD), either in combination with platinum-based therapy or as monotherapy [6], depending on the time frame of disease recurrence. Patients are classified as platinum-resistant and receive monotherapy with the aforementioned agents if disease recurrence occurs in the first 6 months after treatment. Patients are classified as platinum-sensitive and receive platinum-based combination treatment when recurrence occurs >6 months after treatment [7]. A recent study showed that patients with sensitive and resistant EOC show relatively comparable mutation rates [8], suggesting an involvement of epigenetic factors in the resistance development process.

Epigenetic processes are flexible and reversible and provide cells with the ability to adapt to changes in their environment [9]. Cancer cells can hijack this ability by altering epigenetic pathways, providing them with transcriptional plasticity that allows them to evade drug treatment by dysregulation of cellular pathways [10–12]. Indeed, epigenetic resistance mechanisms have been described for several cancer types including ovarian cancer [13–15]. Recently, we performed a chromatin focused multiplexed RNA interference (RNAi) screen, to identify chromatin associated factors that could sensitize EOC cells to platinum-based treatment. We identified subunits of the nucleosome remodelling and deacetylase (NuRD) complex as critical factors sensitizing the EOC cell line A2780 to platinum treatment [16]. In this screen, the FLYWCH-type zinc finger-containing protein 1 (FLYWCH1) was identified as a factor involved in the development of platinum-resistance in EOC cells [16].

So far, the general function of FLYWCH1 and its role in cancer biology is not fully understood. FLYWCH1 was first mentioned in the context of cardiovascular disease, where it was proposed to be a risk factor for coronary artery disease [17] and has been linked to familial mitral valve prolapse [18]. The first publication that focused on FLYWCH1 in cancer postulated that FLYWCH1 directly binds to nuclear β-catenin and thereby inhibits canonical wingless and int- 1 (Wnt) signalling. Through binding to β-catenin, FLYWCH1 selectively blocks the expression of Wnt target genes that influence cell migration and morphology in colorectal cancer (CRC). This suggests not only a tumour suppressive role for FLYWCH1 in CRC but also its potential as a biomarker for or therapeutic target against metastatic CRC [19]. Additionally, FLYWCH1 was shown to negatively regulate canonical Wnt signalling in acute myeloid leukaemia (AML) and to be expressed at low levels in AML [20]. Furthermore, FLYWCH1 was implicated as a potential player in the DNA damage response, where it influences the phosphorylation of γH2A.X which suggests a potential contribution to the recruitment of DNA-damage response proteins [21].

Recent proximity biotinylation studies revealed an interaction of FLYWCH1 with trimethylation of histone 3 on lysine 9 (H3K9me3) and a genome-wide overlap of FLYWCH1 and H3K9me3 in HeLa cells [22]. FLYWCH1 can directly bind to specific DNA binding motifs enriched at (peri)centromeric regions, but it does not directly interact with H3K9me3 peptides. Putative interaction partners of FLYWCH1, identified by mass spectrometry analyses, include H3K9me3-associated proteins, among them methyltransferases, zinc finger proteins, and Polycomb proteins [22]. H3K9me3 is an epigenetic mark predominantly found in heterochromatin, where it is pivotal for genome stability, silencing of repeat elements, and transcriptional gene silencing [23, 24]. Deregulation of repeat element expression is a feature commonly found in different cancer types and other diseases [25–28]. The expression of repeat elements has different effects on the cellular state, strongly depending on the cell and repeat type being expressed. As such, transcription of certain LINE, SINE, and LTR elements leads to the formation of double stranded RNA molecules that induce an immune response and thus have negative effects on cancer development [29, 30]. In contrast, overexpression of the human satellite II (HSATII) element is found in both CRC and EOC, where it is connected to epithelial–mesenchymal transition (EMT) and a poor prognosis for patients [31, 32]. Furthermore, derepression or silencing of different repeat elements contributes to transcriptional plasticity in several ways. On the one hand, members of the short interspersed nuclear element (SINE), long interspersed nuclear element (LINE), and long terminal repeat (LTR) family can act as enhancers and thus derepression positively influences gene expression [33–35]. On the other hand, derepression of certain LTR elements leads to the formation of vast amounts of endogenous retroviruses (ERV) RNAs that can attract transcriptional condensates from actively transcribed genes and lead to their downregulation [36].

In this study we aimed to analyse the role of FLYWCH1 in resistance development of EOC and further characterize its association with H3K9me3 and the resulting gene regulatory function. We found that loss of FLYWCH1 is linked to resistance development in EOC cells and poor prognosis of patients with EOC. FLYWCH1 is localized to H3K9me3 in platinum-sensitive cells, but this colocalization is lost during resistance development. Acute platinum treatment leads to an increase in both FLYWCH1 and H3K9me3 independent of the DNA damage response, hinting at a role for FLYWCH1 in chromatin remodelling. Indeed, loss of FLYWCH1 leads to changes in H3K9me3 in distinct genomic loci, including repeat elements. This induces changes in gene and repeat expression and fosters transcriptional plasticity, which promotes the resistant phenotype. Taken together, our findings provide new insights into the molecular functions of FLYWCH1 in chromatin biology and its significance in platinum resistance development. Our data suggest FLYWCH1 as a potential novel predictive biomarker for the treatment response of patients with EOC.

## Materials and methods

### Plasmids

The shRNAs were cloned into the SGEN vector (pRRL-SFFV-green fluorescent protein (GFP)-miRE-PGK-NeoR) and the LT3GEN vector (pRRL-TRE3G-GFP-miRE-PGK-NeoR) [37]. Guide sequences of the small hairpin RNAs (shRNAs) are described in Supplementary Table S1.

For transfection experiments, the nuclear localization signal (NLS) of SV40 or FLYWCH1 was cloned into the mVenus C1 vector (CMV-mVenus, gift from Steven Vogel, Addgene #27794) [38] together with an 18AA linker to generate empty-mVenus (CMV-NLS-Linker-mVenus) and FLYWCH1-mVenus (CMV-FLYWCH1-Linker-mVenus). Intrinsically disordered regions (IDRs), IDR1 (AA 1–61) and IDR2 (AA 652–715) of FLYWCH1, were cloned into empty-mVenus vector to generate FLYWCH1-IDR1-mVenus (CMV-FLYWCH1-IDR1-Linker-mVenus) and FLYWCH1-IDR2-mVenus (CMV-FLYWCH1-IDR2-Linker-mVenus). CBX3 was cloned into CMV-2xHP1CD-Linker-dsRed [39] to generate CBX3-dsRed (CMV-CBX3-Linker-dsRed).

For overexpression experiments, 3XFLAG or FLYWCH1 and 3XFLAG were cloned into the pRRL-TRE3G-P2A-EGFP-PGK-BLASTI vector to generate pRRL-TRE3G-3XFLAG-P2A-EGFP-PGK-BLASTI and pRRL-TRE3G-FLYWCH1-3XFLAG-P2A-EGFP-PGK-BLASTI.

For protein expression and purification, dsRed and mVenus were cloned into the pET-28a(+) expression vector (Sigma–Aldrich, 69864) to generate pET-dsRed-His and pET-mVenus-His vectors. CBX3 was cloned into the pET-dsRed-His vector and the second IDR of FLYWCH1 (AA 652–715) into the pET-mVenus-His vector to generate pET-CBX3-dsRed-His and pET-FLYWCH1-IDR2-mVenus-His.

The core components for the reporter assay (reporter cassette: pMSCV-tetO-EF1a-mCherry-2A-Blasti, positive control effector: pRRL-rTetR-CBX3-P2A-Hygro, negative control effector: pRRL-rTetR-P2A-Hygro) were previously generated in our lab [40]. FLYWCH1 was inserted into the effector plasmid using standard cloning techniques. In addition, TagBFP was cloned into all three effector vectors yielding pRRL-rTetR-FLYWCH1/rTetR-CBX3/rTetR-P2A-Hygro-IRES-TagBFP.

### Antibodies

Anti-FLYWCH1 antibody (Sigma, HPA041001), anti-H3K9me3 antibody (Diagenode, C15200146), anti-γH2A.X (Ser 139) antibody (Santa Cruz Biotechnology, sc-517348), anti-H3K27me2me3 antibody (Active Motif, 39536), anti-E-Cadherin (BD, 610182), anti-mouse IgG isotype control (Thermo Fisher, 31903), anti-rabbit IgG isotype control (R&D, AB-105-C), Goat Anti-Rabbit IgG H&L Alexa Fluor^®^ 488 (Abcam, ab150077), Goat Anti-Mouse IgG H&L Alexa Fluor^®^ 594 (Abcam, ab150116), Donkey anti-Mouse IgG (H + L) Alexa Fluor^™^ Plus 555 (A32773, Thermo Fisher), and Donkey anti-Rabbit IgG (H + L) Alexa Fluor^™^ Plus 647 (A32795, Thermo Fisher).

### Cell lines and cell culture

Cell lines were purchased from Sigma–Aldrich supplied by ECACC or ATTC and authenticated. All media were supplemented with 10% fetal bovine serum, 4 mM L-glutamine, 10 mM HEPES, 1 mM sodium pyruvate solution, 100 U/ml penicillin, and 100 μg/ml streptomycin. A2780, A2780cis, and SKOV3 cells were cultured in supplemented RPMI 1640 medium, Lenti-X 293T, and Platinum-E retroviral packaging cells were cultured in supplemented Dulbecco's Modified Eagle Medium (DMEM). Media of A2780cis cells was additionally supplemented with 1 μM cisplatin (cPt) (Acros organics, 15663-27-1).

Cultivation of patient-derived organoids (PDOs) was conducted as previously published [41, 42]

### Generation of Eco-positive and tet-on competent cells

All cell lines used were modified to express the ecotropic receptor (EcoR) and the rtTA3 transcriptional activator for the Tet-on system using retroviral transduction as recently described [16]. Cell lines used in the reporter assay were modified to only express EcoR. In brief, Lenti-X cells were transfected with the pRRL.RiEP plasmid (pRRL.SFFV-rtTA3-IRES-EcoR-PGK-Puro, gift from Johannes Zuber) or pRRL-EP plasmid (pRRL.SFFV-EcoRec-PGK-Puro) and the two packaging vectors pCMVR8.74 (gift from Didier Trono, Addgene #22036) and pCAG-VSVG (gift from Arthur Nienhuis & Patrick Salmon, Addgene #35616) in a 4:2:1 ratio and with 3× (w/w) excess of polyethyleneimine 25K for virus production. After 40–45 h, viral supernatant was harvested, filtered and target cells were transduced with viral supernatant and selected with Puromycin. Derived cell lines were subsequently transduced with ecotropically packaged retroviruses.

### Lentiviral transduction of cells

Retroviral packaging of shRNA expressing or overexpression vectors was performed as previously described [16]. In brief, Lenti-X cells were transfected with the plasmid of interest and the two packaging vectors pCMVR8.74 and pCAG-Eco (gift from Arthur Nienhuis & Patrick Salmon, Addgene #35617) in a 4:2:1 ratio and with 3× (w/w) excess of polyethyleneimine 25K. After 40–45 h, the viral supernatant was collected, filtered and added to the Tet-on competent target cells at a ratio that leads to a transduction efficiency <30% to ensure single plasmid integration. Retroviral packaging of the reporter cassette was performed as previously described [40]. In brief, PlatE cells were transfected with 20 μg of the plasmid carrying the reporter cassette and 10 μg of GagPol helper DNA. Viral supernatant was collected, filtered and added to the Eco+ target cells at a ratio that leads to a transduction efficiency <30% to ensure single plasmid integration.

### Competitive proliferation assay

SKOV3: Cells were retrovirally transduced with shRNA expressing SGEN plasmids. From day 5 after transduction, the percentage of shRNA-expressing (GFP-positive) cells was measured every 2–3 days using the flow cytometer.

A2780 and A2780cis: Cells were retrovirally transduced with shRNA expressing LT3GEN plasmids or with plasmids for 3xFLAG and FLYWCH1-3XFLAG overexpression. For overexpression assays, cells were selected with 7.5 μg/ml blasticidin and then mixed in a 50/50 ratio with untransduced cells. Knockdown (KD) or overexpression were induced by the addition of 1 μg/ml doxycycline (Dox), and the percentage of construct-expressing (GFP-positive) cells was measured every 2–3 days using the flow cytometer. After 7 days of overexpression or KD induction, the cell population was split and half the cells treated with 1 μM cPt until the end of the assay.

### Reporter assay

The reporter assay was performed as recently described [40]. In brief, A2780 and A2780cis cells were retrovirally transduced with virus containing the reporter cassette and selected with 5 μg/ml blasticidin for 4 days. When cells were fully recovered, they were retrovirally transduced with virus containing the effector constructs carrying TagBFP and selected with 500 μg/ml hygromycin for 4 days. Cells were split onto two wells and either treated with vehicle or 1 μg/ml Dox to recruit effectors to the synthetic promotor. Reporter expression in vehicle and Dox treated BFP+ cells was analysed every 2–3 days by using the flow cytometer.

### Gene-expression analysis

To validate the KD of FLYWCH1, shRNA expression was induced for 7 or 10 weeks (A2780 or SKOV3). One week before the end of the assay, the selection of cells was started with 2 or 3 mg/ml (SKOV3 or A2780) G418 solution for 7 days. Cells were then harvested or if necessary sorted for shRNA expressing/GFP-positive cells. RNA extraction was performed using the RNeasy Plus Mini Kit (QIAGEN, 74134), and messenger RNA (mRNA) was transcribed into complementary DNA (cDNA) using MultiScribe^™^ Reverse Transcriptase (Thermo Fisher Scientific, 4311235). Gene expression was then determined by real-time quantitative polymerase chain reaction (RT-qPCR) using the ORA^™^ SEE qPCR Green ROX L Mix (highQu, QPD0505) and the CFX Real-Time PCR detection system (Bio–Rad). Succinate dehydrogenase (SDHA) expression was used for normalization. Primers used for RT-qPCR are described in Supplementary Table S2.

To analyse the expression of FLYWCH1 in PDO cell lines, two wells of fully grown organoids were harvested, and RNA was isolated using the RNeasy Mini Kit (QIAGEN, 74104). Reverse transcription to cDNA was performed using the cDNA Synthesis Kit (Biozym, 331470X). Gene expression was determined by RT-qPCR on an Applied Biosystems^™^ 7500 Fast Real-Time PCR System using the TaqMan System for FLYWCH1 (Hs00946896_mH) and glyceraldehyde 3-phosphate dehydrogenase (GAPDH, Hs99999905_m1). GAPDH expression was used for normalization.

### Patient cohort and ethics approval

PDOs were generated as part of a biobanking process [41, 42], with the approval of the Ethics Commission of LMU University, and with the written consent of the patients. Lines included in the study originated from primary cancer tissue obtained during debulking surgeries of chemotherapy naïve patients.

The patient cohort used for immunohistochemistry is well characterized and has been described before [43, 44]. Specimens used in this paper represent a cohort of 155 patients with EOC [serous (*n* = 109), endometrioid (*n* = 21), clear cell (*n* = 12), and mucinous (*n* = 13)) who underwent radical cytoreductive surgery at the Department of Obstetrics and Gynaecology of the LMU University Hospital between 1990 and 2002. Histopathological diagnoses were established by specialized gynaecologic pathologists with staging and grading according to TNM and FIGO (International Federation of Gynaecology and Obstetrics) classification. 75.2% of patients presented with advanced disease (FIGO IIB-IV), while only 24.8% were diagnosed in early disease (FIGO I-IIA). Except for patients in stage FIGO IA with low-grade histology, all patients received first-line platinum-based chemotherapy. Lifetime data (birth, primary EOC diagnosis, relapse, death) from EOC patients were taken from our patient charts, the Munich Cancer Registry and aftercare calendars. Median age at primary diagnosis was 59.0 years with a 95% confidence interval (CI) of 57.0–61.0 years. 28 relapses and 101 deaths were documented. This study has been approved by the ethics committee of Ludwig Maximilian University of Munich (reference numbers: 138/03 and 17/-0471) and was carried out in compliance with the guidelines of the Helsinki Declaration of 1964 (last revision: October 2018). All participants gave their written informed consent. Samples and clinical information were anonymized for statistical workup.

### Tissue microarrays and immunohistochemistry

Tissue microarrays and immunohistochemistry (IHC) have been performed as previously described [16, 43]. In brief, representative regions with a diameter of 0.6 mm were taken of the paraffin-embedded tumour samples biopsies and arrayed into a recipient paraffin block (30 × 20 × 10 mm) by using a micro tissue arrayer (Beecher Instruments, Sun Prairie, WI, USA). Every tumour sample was used for three biopsies, resulting in 465 tissue micro assays in total. Sections of 5 μm were prepared and transferred to microscope slides. Haematoxylin and eosin staining were performed to ensure that there was enough representative tumour tissue left. IHC using the anti-FLYWCH1 antibody was performed using a combination of pressure cooker heating and the ZytoChem-Plus HRP Polymer-Kit with DAB as chromogenic substrate as previously described [45]. Evaluation, imaging and storing was done with an AxioScope microscope (Carl Zeiss, Jena, Germany), an AxioCam digital camera system (Carl Zeiss) and the AxioVision software (Carl Zeiss). Immunohistochemical staining was assessed semi-quantitatively as previously described [46] by using the IHC score (mean ± SEM). Mean values of the three representative IHC scores of every probe were calculated for further analysis.

### Transfection of cells for ectopic expression of FLYWCH1

For transfections with one construct, 3–6 × 10^5^ A2780 cells were seeded in 6-wells onto microscopy coverslip coated with poly-L-lysine (Sigma–Aldrich, P4707). When cells were reattached (after 6–8 h), they were transfected with 200 ng of the plasmid of interest and 1.3 μg of filler DNA using FuGENE HD (Promega, E2311) in a ratio of 3:1 (FuGENE:DNA). Media was exchanged after 24 h and after a further 24 h of recovery cells were stained for H3K9me3 or IgG isotype control.

For transfection with two constructs, 4–8 × 10^5^ A2780 cells each were seeded in 6-wells onto microscopy coverslip coated with poly-L-lysine (Sigma–Aldrich, P4707). The next day, cells were transfected with 1.5 μg of mVenus vector (empty-mVenus, FLYWCH1-mVenus, FLYWCH1-IDR1-mVenus, or FLYWCH1-IDR2-mVenus), 1.5 μg of dsRed vector (CBX3-dsRed or empty-dsRed gift by Anja R. Köhler), 2 μg of filler DNA, and 7.5 μl Lipofectamine^™^ 3000 Reagent (Thermo Fisher Scientific, L3000015). After a recovery period of 8 h, when cells showed first signs of fluorescence, media was changed for fresh media containing either vehicle or 1 μM cPt. After a further 24 h cells were prepared for imaging. Washing steps were always performed three times for 5 min with PBS^Mg2+ Ca2+^ (Sigma–Aldrich, D8662). Cells were washed and then fixed with 1 ml of 4% paraformaldehyde (Thermo Fisher Scientific, 28908) in PBS for 10 min at room temperature. Cells were washed with PBS and then cell nuclei stained with 1 μg/ml of DAPI (4',6-diamidino-2-phenylindole) (Sigma–Aldrich, D9542) 1:5000 in PBS^Mg2+ Ca2+^ for 3 min at room temperature in the dark. Cells were washed again with PBS and then mounted onto a microscopy slide using Mowiol^®^ 4–88 (Sigma–Aldrich, 81381).

### Immunofluorescence staining

A2780 cells were pretreated with 1 μM cPt for 1–6 days. The day before the immunostaining, 4 × 10^5^ cells per condition were seeded into 6-wells onto poly-L-lysine (Sigma–Aldrich, P4707) coated microscopy coverslips. Treatment was continued for 24 h and after a total of 1–7 days treatment, immunostaining was performed. All washing steps were performed three times for 5 min with PBS^Mg2+ Ca2+^ (Sigma–Aldrich, D8662). Cells were washed and then fixed with 1 ml of 4% paraformaldehyde (Thermo Fisher Scientific, 28908) in PBS for 10 min at room temperature. Cells were washed with PBS and then permeabilized with 1 ml of ice cold 0.3% Triton X-100 (Sigma–Aldrich, X100) in PBS^Mg2+ Ca2+^ for 5 min at 4°C. Cells were washed again with PBS and then incubated shaking in blocking solution [5% (w/v) BSA in PBS^Mg2+ Ca2+^] for 1 h at room temperature. Following blocking, the cells were stained with primary antibody anti-FLYWCH1 (1:150), anti-H3K9me3 (1:500), anti-γH2A.X (1:100), anti-H3K27me2me3 (1:300), or anti-mouse IgG isotype control (1:1500) diluted in 5% BSA in PBS^Mg2+ Ca2+^ at 4°C overnight. Cells were then washed with PBS and subsequently stained with secondary antibody Goat Anti-Rabbit Alexa Fluor^®^ 488 (1:1000) and Goat Anti-Mouse Alexa Fluor^®^ 594 (1:1000) in 5% BSA in PBS for 2 h at room temperature in the dark. Cells were washed again with PBS and then cell nuclei stained with 1 μg/ml DAPI (Sigma–Aldrich, D9542) in PBS^Mg2+ Ca2+^ for 3 min at room temperature in the dark. Cells were washed again with PBS and then mounted onto microscopy slides using Mowiol^®^ 4–88 (Sigma–Aldrich, 81381).

For immunostaining of PDO cell lines, two fully grown wells of high-grade serous ovarian cancer organoids were fixed as previously described [41] in 4% Paraformaldehyde (PFA), and centrifuged at 100g to avoid fragmentation. PDOs were blocked and permeabilized with 4% BSA, 0.05% Tween, and 0.5% Triton-X100 in PBS overnight at room temperature. The next day, primary antibody anti-FLYWCH1 (1:150), anti-E-Cadherin (1:200), or anti-H3K9me3 (1:200) were added to the cells and incubated overnight at 4°C. Following secondary antibody incubation with Donkey anti-Mouse Alexa Fluor^™^ Plus 555 and Donkey anti-Rabbit Alexa Fluor^™^ Plus 647 (1:1000) for 2 h, cells were stained with Phalloidin AF488 (0.165 μM diluted in Blocking solution, Invitrogen) for 1 h. After nuclear staining with DAPI (1:1000, Thermo Fisher, 62248), the PDOs were mounted using Mowiol.

### Image acquisition and analysis

Images for PDO lines were acquired on a Leica SP8 confocal microscope equipped with a HC PL APO CS2 40×/1.30 OIL objective and the following excitation wavelength (ex.) and emission windows (em.): DAPI ex. 405 nm/em. 415–480 nm, Phalloidin AF488 ex. 490 nm/em. 501–535 nm, Alexa Fluor^™^ Plus 555 ex. 550 nm/em. 560–615 nm, and Alexa Fluor^™^ Plus 647 ex. 645 nm/em. 655–710 nm. Image of PDO lines were processed with the LAS X software. For better visualization of single cells, images were cropped in the LAS X software to achieve digital zoom.

Images of transfections and immunostainings of cells lines were acquired using a Zeiss LSM 710 Laser Scanning Confocal Microscope equipped with a Plan-Apochromat 63×/1.4 Oil DIC M27 objective. Excitation wavelength and emission window in immunostainings were chosen as follows: DAPI ex. 405 nm/em. 410–507 nm, Alexa Fluor^®^ 488 ex. 488 nm/em. 496–611 nm, Alexa Fluor^®^ 594 ex. 561 nm/em. 585–733 nm, mVenus ex. 514 nm/em. 515–553 nm, and dsRed ex. 561 nm/em. 566–703 nm. Lasers and detection wavelength were kept the same for all samples within a replicate. Images of the whole nucleus were taken by acquisition of Z-stacks with an interval of 0.5 μm. Generation of maximum intensity projections (MIPs) and image processing were performed with ZEN blue and black software version 2.5 (Zeiss). For better visualization of single cells and foci, images were cropped in the ZEN blue software to achieve digital zoom. Quantitative analysis of number of foci, foci area and staining intensity in the nucleus was performed on MIPs, correlation analysis on single focal plane images. All quantitative analysis was performed using CellProfiler version 4.2.6 [47]. Profile analysis was performed using the Plot Profile function in Image J 2.14.0/1.54f [48].

### Protein expression and purification

For purification of His-tagged dsRed, mVenus, CBX3-dsRed, and FLYWCH1-IDR2-mVenus, *Eschrichia coli* BL21-CodonPlus (DE3)-RIL cells were transformed with 50 ng of the respective plasmids using heat-shock and incubated overnight on LB-agar supplemented with 35 μg/ml chloramphenicol and 50 μg/ml kanamycin (selection LB).

The following day, 20 ml of selection LB media were inoculated using one colony and incubated for 6 h at 37°C and 150 rpm. 500 ml of selection LB media was inoculated with 5 ml of starter culture and cultivated at 37°C and 150 rpm until OD_600_ reached 0.6–0.8. Protein expression was induced by addition of 500 μM isopropyl β-D-1-thiogalactopyranoside (IPTG), and the culture was incubated for 14 h at 17°C and 150 rpm. Cell were harvested at 5000 × *g*, 4°C for 15 min, washed once with STE buffer (100 mM NaCl, 10 mM Tris HCl pH 8, 1 mM EDTA) and stored at −20°C until purification.

For purification, cell pellets were resuspended in 30 ml of sonication buffer (30 mM KPI-buffer pH 7.2, 0.2 mM dithiothreitol (DTT), 500 mM KCl, 1 mM EDTA, 10% glycerol, 20 mM Imidazole) with protease inhibitor and cells lysed by sonication for 5 min (15 s pulses, 30 s pauses, 90% amplitude) using an EpiShear Probe Sonicator (Active Motif). The cell lysate was cleared by centrifugation for 1 h at 20 000 rpm and filtered through a 0.45 μM CHROMAFIL GF/PET-45/25 filter (MACHEREY-Nagel). Affinity chromatography was performed using purification columns filled with Ni-NTA superflow beads (Clontech) and an NGC FPLC system (Bio–Rad). Proteins were eluted using elution buffer (30 mM KPI buffer pH 7.2, 500 mM KCl, 1 mM EDTA, 0.2 mM DTT, 10% glycerol, and 220 mM imidazole). Dialysis was performed for 2 h at 8°C to transfer proteins into dialysis buffer (20 mM HEPES pH 7.2, 200 mM KCl, 1 mM EDTA, 0.2 mM DTT, and 10% glycerol). Proteins were aliquoted, snap-frozen and stored at −80°C.

### Co-compartmentalization assay

To investigate co-compartmentalization of FLYWCH1 and CBX3, 2 μM of mVenus or FLYWCH1-IDR2-mVenus were incubated with 2 μM dsRed or CBX3-dsRed in crowding buffer (40 mM Tris–HCl pH 7.5, 20% PEG). Total amount of dialysis buffer in the reaction was kept constant, leading to a total composition of the reaction of 4 μM total protein, 40 mM Tris–HCl pH 7.5, 20% PEG, 3 mM HEPES, 30 mM KCl, 0.15 mM EDTA, 0.03 mM DTT, and 0.015% glycerol. After 20 min of incubation at room temperature, 5 μl of sample were transferred onto a microscopy slide and the coverslip was placed carefully from left to right. Samples were incubated for a further 5 min until movement by capillary action decreased and droplets were imaged using a Zeiss Cell Observer Z1 epifluorescence microscope equipped with a Plan-Apochromat 63×/1.4 Oil DIC M27 objective. Excitation wavelength and emission window were chosen as follows: mVenus ex. 495 nm/em. 500–550 nm and dsRed ex. 570 nm/em. 570–640 nm. Image processing were performed with ZEN blue and black software version 2.5 (Zeiss).

### Intracellular antibody staining

To investigate changes in H3K9me3 levels upon platinum treatment, A2780 cells were treated with 1 μM cPt for 1–7 days. To investigate changes in H3K9me3 levels upon FLYWCH1 KD, shRNA expression was induced for 7 weeks. One week before the end of the assay, selection of cells was started with 3 mg/ml G418 solution for 7 days. Cells were harvested, washed with PBS^Mg2+ Ca2+^ and 1–1.5 × 10^6^ cells per condition transferred to a 96-well U-bottom plate. Cells were pelleted at 300 × *g* for 5 min and fixed in 100 μl of 4% PFA (Thermo Fisher Scientific, 28908) for 15 min at room temperature. All further washing steps were performed by adding excess PBS^Mg2+ Ca2+^ to the cells followed by centrifugation at 600 × *g* for 5 min. After washing, cells were incubated in 100 μl of cell permeabilization buffer (0.5% BSA and 0.3% Triton X-100 in PBS^Mg2+ Ca2+^) for 10 min at room temperature. Cells were washed and then blocked by incubation with 100 μl of 5% BSA in PBS^Mg2+ Ca2+^ for 1 h at room temperature. Following another wash step, each pellet was split onto two wells with 0.5–0.75 × 10^6^ cells each. Pellets were resuspended in primary antibody (180 ng anti-H3K9me3 antibody) or isotype control (180 ng anti-mouse IgG) in antibody dilution buffer (0.5% BSA in PBS^Mg2+ Ca2+^) and incubated for 1 h at room temperature. Following another wash step, cells were resuspended in 100 μl of secondary antibodies (Goat Anti-Mouse Alexa Fluor® 594) diluted 1:500 in antibody dilution buffer. Cells were incubated protected from light for 30 min at room temperature and washed one last time. Cells were then resuspended in 150 μl of PBS and analysed by using the flow cytometer. To determine changes in H3K9me3 levels, H3K9me3 signal was background corrected by subtracting the signal of the isotype control.

### RNA-seq

For RNA-seq in FLYWCH1 KD cells, A2780 cells were retrovirally transduced with shRNA-expressing plasmids. KD was induced by the addition of 1 μg/ml Dox. After 1 week of KD induction, cells were either treated with vehicle or 1 μM cPt. After 6 weeks of further KD induction, cells were selected with 3 mg/ml G418 solution for one week and after a total of 7 weeks of KD induction they were harvested or if necessary sorted for shRNA-expressing/GFP-positive cells. For RNA-seq in A2780cis cells with reintroduction of FLYWCH1, A2780cis cells were retrovirally transduced with 3XFLAG (control) or FLYWCH1-3XFLAG expression vectors. After 7 days of induced overexpression via the addition of 1 μg/ml Dox, cells were harvested and sorted for construct-expressing/GFP-positive cells. RNA extraction was performed using the RNeasy Plus Mini Kit (QIAGEN). The RNA-seq libraries were prepared using 100 ng of RNA and the NEBNext^®^ Single Cell/Low Input RNA Library Prep Kit for Illumina^®^ (NEB, E6420) and NEBNext^®^ Multiplex Oligos for Illumina^®^ (Dual Index Primers Set 1) (NEB, E7600) kit. Sequencing of the RNA-seq libraries was performed by Novogene on an Illumina NovaSeq 6000 deep sequencer acquiring 2 × 150 bp paired-end reads aiming at 10 mio paired-end reads (20 mio total reads) per sample.

### Analysis of RNA-seq data

Preparation: RNA-Seq data were processed using the galaxy web platform (https://usegalaxy.eu/) [49]. Quality of sequencing data was assessed using FastQC (Galaxy Version 0.74 + galaxy0) [50] and MultiQC (Galaxy Version 1.11 + galaxy1) [51]. Low quality bases and adapter contaminations were trimmed using Trim Galore! (Galaxy Version 0.6.7 + galaxy0) [52].

Gene expression analysis: The quality controlled and trimmed sequences were mapped to the human reference genome hs1 (T2T CHM13v2.0) using HISAT2 (Galaxy Version 2.2.1 + galaxy1) [53]. The number of reads per gene was assessed using featureCounts (Galaxy Version 2.0.3 + galaxy2) [54] and differential gene expression analysis was performed using DESeq2 (Galaxy Version 2.11.40.8 + galaxy0) [55]. DeSeq2 outputs are available in Supplementary Tables S4–S6 and S8. Differentially expressed genes (DEGs) were filtered to identify groups with different and similar effects. *Z*-scores were calculated in excel and heatmaps for the differential groups plotted using the R package ComplexHeatmap [56]. Venn diagrams were created using the web application DeepVenn [57]. Alluvial plots were generated using the R package alluvial [58]. Pathway analysis was performed in the GSEA software version 4.3.2 [59] using normalized counts from DeSeq2, including only genes with a mean count of ≥ 1 per sample.

Repeat expression analysis: To analyse expression changes in repeat elements, the quality controlled and trimmed sequenced were mapped to the human reference genome hs1 (T2T CHM13v2.0) using RNA STAR (Galaxy Version 2.7.11a + galaxy0) [60], while allowing for multimapping of reads (–outFilterMultimapNmax 100). The number of reads per repeat element was assessed using a previously published GTF file containing annotations of the known repeat elements [61] and the package featureCounts (Galaxy Version 2.0.3 + galaxy2) [54], while allowing reads to map to multiple features (-M -O). Differential repeat expression analysis was performed using DESeq2 (Galaxy Version 2.11.40.8 + galaxy0) [55]. DeSeq2 outputs are available in Supplementary Tables S11–S14. Heatmaps of repeat expression were generated in Tableau version 2022.2.2 (Salesforce, San Francisco, CA, USA).

### Chromatin immunoprecipitation sequencing (ChIP-seq)

For chromatin immunoprecipitation sequencing (ChIP-seq) of sensitive and resistant cells, 2.5 × 10^6^ cells were harvested and washed with PBS once. For ChIP-seq of FLYWCH1 KD, A2780 cells were retrovirally transduced with shRNA-expressing plasmids and KD was induced by the addition of 1 μg/ml Dox for a total of 7 weeks. Treatment with vehicle or 1 μM cPt was started 1 week after KD induction. After 6 weeks of KD induction, cells were selected with 3 mg/ml G418 solution and after a total of 7 weeks of KD induction portions of 2.5 × 10^6^ cells were harvested or if necessary sorted for shRNA-expressing/GFP-positive cells and were washed once PBS. Cells were lysed in 125 μl lysis buffer (10 mM Tris–HCl pH 7.4, 2 mM MgCl_2_, 0.6% Igepal-Nonidet P40, 0.5 mM phenylmethylsulfonyl fluoride, 1 mM DTT, cOmplete™ EDTA-free protease inhibitor cocktail) for 15 min on ice. Samples were digested using 300 U of micrococcal nuclease for 12.5 min at 37°C, then samples were put on ice and the reaction stopped by addition of 8.8 μM EDTA, 0.08% Triton X-100 and 0.08% sodium deoxycholate solution. Samples were vortexed briefly, incubated on ice for 15 min, vortexed extensively for 30 s and then diluted with 800 μl Complete IP buffer (20 mM Tris–HCl pH 8.0, 2 mM EDTA, 150 mM NaCl, 0.1% Triton X-100, 1 mM phenylmethylsulfonyl fluoride, cOmplete^™^ EDTA-free protease inhibitor cocktail). Samples were incubated for 1 h at 4°C with rotation and then centrifuged at 13 000 × *g* for 10 min at 4°C to pellet remaining cellular debris. For the preparation of antibody–bead complexes, 25 μl of Dynabeads^™^ Protein G (Thermo Fisher Scientific, 10004D) per sample were washed with Complete IP buffer three times and then resuspend in 200 μl complete IP buffer and 5 μg of anti-H3K9me3 or anti-mouse IgG, respectively. Bead were incubated with the antibodies for 3 h at 4°C at constant rotation to allow the formation of antibody–bead complexes. For the preclearing of chromatin, 25 μg of chromatin for each sample were mixed with 5% mononucleosomes isolated from Drosophila melanogaster as spike-in control (1.25 μg). Chromatin samples were precleared by incubation with 25 μl Dynabeads^™^ Protein G (Thermo Fisher Scientific, 10004D) and 2.5 μg anti-mouse immunoglobulin G (IgG) for 3 h at 4°C and constant rotation. Beads containing unspecifically bound chromatin were removed from the precleared samples using a magnetic rack and 10% of each sample was saved as input. Precleared chromatin was then added to the antibody–bead complexes. Chromatin and beads were incubated overnight at 4°C with rotation. The next day, unbound chromatin in the supernatant was discarded and the beads with bound chromatin were washed twice with low salt wash buffer (20 mM Tris–HCl pH 8.0, 2 mM EDTA, 150 mM NaCl, 1% Triton X-100, 0.1% sodium dodecyl sulfate) and twice with high salt wash buffer (20 mM Tris–HCl pH 8.0, 2 mM EDTA, 500 mM NaCl, 1% Triton X-100, 0.1% sodium dodecyl sulfate) for 10 min at 4°C. Subsequently, 200 μl of ChIP elution buffer (100 mM NaHCO_3_, 1% sodium dodecyl sulfate) was added to the beads and the saved input samples together with 2 μg RNase A (Machery-Nagel, 740 505) and incubated for 30 min at 37°C. Then, 2.4 U Proteinase K (NEB, P8107S) were added and samples were incubated for 2 h at 65°C with constant shaking. After this, DNA in the supernatant was purified using the Chromatin IP DNA Purification Kit (Active Motif, 58002). ChIP libraries were prepared using the NEBNext^®^ Ultra^™^ II DNA Library Prep Kit for Illumina^®^ (NEB, E7103) and the NEBNext^®^ Multiplex Oligos for Illumina^®^ (Dual Index Primers Set 1) (NEB, E7600). Sequencing of the ChIP-seq libraries was performed by Novogene on an Illumina NovaSeq 6000 deep sequencer acquiring 2 × 150 bp paired-end reads aiming at 20 mio paired-end reads (40 mio total reads) per sample.

### Analysis ChIP-seq data

Preprocessing of ChIP-seq data: ChIP-seq data were analysed using the galaxy web platform (https://usegalaxy.eu/) [49]. Quality of sequencing data were assessed using FastQC (Galaxy Version 0.74 + galaxy0) [50]. Adapter contaminations were removed using BBTools:BBduk (Galaxy Version 1.0.0 + galaxy3) [62], and low quality bases were trimmed using Trimmomatic (Galaxy Version 0.38.1) [63]. The quality controlled and trimmed sequences were mapped to the human reference genome hs1 (T2T CHM13v2.0) using HISAT2 (Galaxy Version 2.2.1 + galaxy1) [53], and PCR duplicates detected and removed by MarkDuplicates (Galaxy Version 2.18.2.3) function of Picard [64] and RmDup (Galaxy Version 2.0.1) function of samtools [65]. The ChIP-seq data were then binned into genomic intervals of 200 bp using Samtools bedcov (Galaxy Version 2.0.3) [65] and subsequently quantile normalized on the bwUniCluster using a recently published python script (https://github.com/Rathert-lab) [16]. Input samples and IP samples were normalized separately. Quantile normalized bedgraph files were converted into bigwig files using Wig/BedGraph-to-bigWig (Galaxy Version 1.1.1).

Peak-calling and identification of differential peaks: Peaks in the ChIP-seq data were identified using the MACS2 package [66] on galaxy. First, noise reduction was performed by subtracting the signal in the input samples from the signal in the IP samples using MACS2 bdgcmp (Galaxy Version 2.2.7.1 + galaxy0). Next, broad peaks were called from noised reduced data using MACS2 bdgbroadcall (Galaxy Version 2.2.7.1 + galaxy0) with a cut-off for peaks of 1000/850 and a cut-off for linking regions of 500/425 for samples from FLYWCH1 KD/sensitive and resistant cells, respectively. BED files were further processed using the bedtools suite [67] on galaxy. To analyse all peaks in the respective dataset (FLYWCH1 KD or sensitive/resistant cells), peaks were concatenated using Concatenate datasets (Galaxy Version 0.1.1). After sorting of the BED files using bedtools SortBED (Galaxy Version 2.30.0 + galaxy2), nearby and overlapping intervals were merged using bedtools MergeBED (Galaxy Version 2.30.0). Finally, nearby and overlapping intervals were clustered using bedtools ClusterBed (Galaxy Version 2.30.0). These two BED files (FLYWCH1 KD or sensitive/resistant cells) were used to identify differential regions in the noise reduced bigwig files using the k-means clustering algorithm in ChAsE version 1.12 [68]. Heatmaps were generated using the quantile normalized bigwig files and plotted with a 10 kb window in 50 bins around the peak centre. Visualization of ChIP-seq tracks was done using the Integrative Genomics Viewer (IGV) version 2.13.2 [69].

Repeat analysis: The RepeatMasker BED file (v4.1.2p1.2022Apr14) containing all repeat elements in the hs1 genome was retrieved from github (https://github.com/marbl/CHM13). Gene annotations for CHM13_T2T_v2.0 were downloaded from NCBI (GCF_009914755.1) and filtered for genes and transcripts. A Promotor BED file was generated by extracting the region 1 kb around the transcription start site and duplicates were removed. Repeat and promotor association of the ChIP-seq peaks was identified by mapping the differential regions against the promotor and RepeatMasker BED using bedtools AnnotateBed (Galaxy Version 2.31.1).

To analyse changes in H3K9me3 signal at repeat elements, the BED files of the differential regions were randomly redistributed across the entire genome three times using bedtools ShuffleBed (Galaxy Version 2.31.1 + galaxy0). The overlaps of the differential peak files and the shuffled files with the RepeatMasker BED was assessed using bedtools AnnotateBed (Galaxy Version 2.31.1) and filtered to contain only repeats with overlaps in at least one of the files. For each repeat class or subfamily, the total number of associated differential peaks (observed) and shuffled peaks (expected by random chance) was determined and the Obs/Exp ratio calculated. For the identification of repeat elements with differential H3K9me3 signal in ChAsE, two BED files were produced from the RepeatMasker BED by selecting all lines containing LTR or SINE elements using the Select (Galaxy Version 1.0.4) function on galaxy. To reduce the total number of regions, all LTR and SINE elements with no H3K9me3 signal were identified using bedtools AnnotateBed (Galaxy Version 2.30.0) and remove from the BED files with the Select (Galaxy Version 1.0.4) function. Differential repeat elements were then identified in ChAsE as explained above.

### Statistical analysis

Statistics of IHC and survival data were determined by using SPSS Statistics 29 (IBM, Chicago, IL, USA). All other statistical analysis was performed in GraphPad Prism version 8.0.2 (GraphPad Software, Boston, MA, USA).

## Results

### Loss of FLYWCH1 promotes platinum-resistance development in EOC cell lines

**Figure 1.**
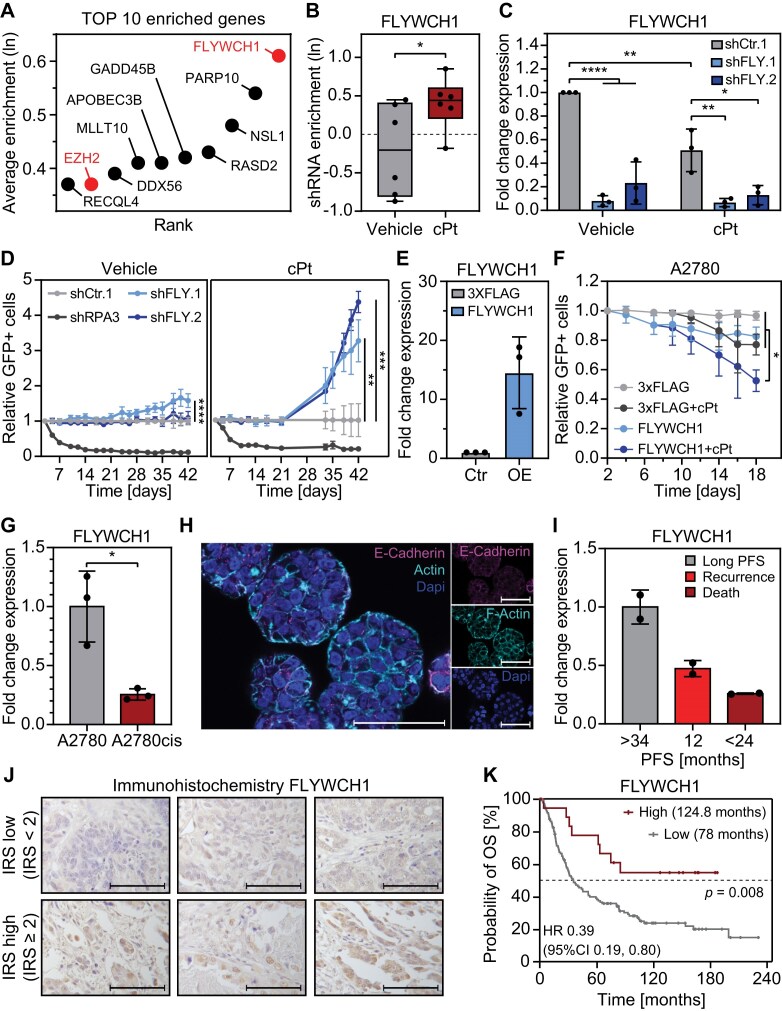
Loss of FLYWCH1 promotes resistance to cPt treatment and a poor prognosis for patients. (**A**) Average enrichment of the top 10 targeted genes enriched under cPt treatment compared to vehicle control from the previously published chromatin-focused RNAi screen [16]. Genes with significant enrichment (*P* ≤ 0.05) are highlighted. Statistical analysis: paired *t*-test. (**B**) Enrichment of shRNAs targeting FLYWCH1 (*n* = 6) in cells treated with either 1 μM cPt or vehicle. Enrichment of shRNAs was calculated relative to shRNAs in the original pools. Average enrichment of each shRNA is shown as a box plot with all data points displayed, *n* = 3. Statistical analysis: paired *t*-test (*P ≤ 0.05). (**C**) Gene expression of FLYWCH1 (RT-qPCR) relative to SDHA control and shCtr.1. FLYWCH1 KD was induced in A2780 cells for 7 weeks with two independent shRNAs targeting FLYWCH1, and cells were treated with vehicle or 1 μM cPt for 6 weeks. Mean ± SD, *n* = 3, statistical analysis: two-way ANOVA and Tukey’s multiple comparisons test (*P ≤ 0.05, **P ≤ 0.01, ****P ≤ 0.0001). (**D**) Competitive proliferation assays of A2780 cells expressing the indicated shRNAs to determine the effects of suppression of FLYWCH1 expression on cell proliferation. Cells were cultivated in the presence of vehicle or 1 μM cPt from day 7 until the end of the assay. Shown is the fraction of shRNA+/GFP+ cells relative to the initial measurement and shCtr.1. Mean ± SD, *n* = 3, statistical analysis: two-way ANOVA and Dunnett’s multiple comparisons test (**P ≤ 0.01, ***P ≤ 0.001, ****P ≤ 0.0001). (**E**) Gene expression of FLYWCH1 (RT-qPCR) relative to SDHA control and 3XFLAG. Expression of FLYWCH1 or 3XFLAG was induced in A2780 cells for 7 days. Mean ± SD, *n* = 3. (**F**) Competitive proliferation assays of A2780 cells expressing 3XFLAG or FLYWCH1 to determine the effects of FLYWCH1 overexpression on cell proliferation. Cells were cultivated in the presence of vehicle or 1 μM cPt from day 7 until the end of the assay. Shown is the fraction of shRNA+/GFP+ cells relative to the initial measurement. Mean ± SD, *n* = 3, statistical analysis: two-way ANOVA and Tukey’s multiple comparisons test (*P ≤ 0.05). (**G**) Gene expression of FLYWCH1 (RT-qPCR) in platinum-sensitive A2780 and resistant A2780cis cells relative to SDHA control and A2780 cells. Mean ± SD, *n* = 3, statistical analysis: unpaired *t*-test with Welch’s correction (*P ≤ 0.05). (**H**) Representative immunofluorescence images of EOC PDOs stained with the indicated antibodies; scale bar: 50 μm, *n* = 2. (**I**) Gene expression of FLYWCH1 (RT-qPCR) in three EOC PDOs derived from different patients with variable PFS times and treatment outcomes. Mean ± SD, *n* = 3. (**J**) Representative immunohistochemistry images of EOC patient tissue stained against FLYWCH1. Images were evaluated by the IRS to obtain a binary classification of the expression for the Kaplan–Meier survival analysis; scale bar: 100 μm. (**K**) Kaplan–Meier survival analysis for OS of 155 patients with EOC with low (IRS < 2) or high (IRS ≥ 2) FLYWCH1 expression before treatment with chemotherapy. Median survival of each group is indicated. Statistical analysis: Chi-Square statistics of the log-rank test (Mantel-Cox).

We previously performed a multiplexed RNAi screen to identify chromatin associated factors that affect the platinum response in EOC cells. In this screen, we identified coregulators that sensitize EOC cells to treatment with platinum-based chemotherapy and prevent the development of drug resistance [16]. In order to identify chromatin associated cofactors that are instead involved in the resistance development process, we filtered the data from this screen for targets that show enrichment upon cPt treatment and ranked them by their average enrichment. Among the top 10 hits, loss of FLYWCH1 and EZH2 were the only ones leading to significant enrichment upon cPt treatment (Fig. 1A). A2780 cells with loss of FLYWCH1 or EZH2 induced via six independent shRNAs showed a significant enrichment upon cPt treatment compared to untreated cells (Fig. 1B and Supplementary Fig. S1A). To validate the effect of FLYWCH1 loss observed in the screen, we performed single KD validation experiments in two different EOC cell lines and with two independent shRNAs targeting FLYWCH1. After one week of KD induction, cells were treated with vehicle or cPt and cultivated until proliferation resumed in the presence of cPt, at which point they had gained early resistance to the treatment (Supplementary Fig. S1B). Interestingly, early resistance development (shCtr.1 + cPt) led to a significant decrease in FLYWCH1 expression (Fig. 1C). Loss of FLYWCH1 only induced a mild proliferative advantage of vehicle treated cells (Fig. 1D, left panel, and Supplementary Fig. S1C and D). On the contrary, loss of FLYWCH1 led to a significant increase in proliferation in cPt treated platinum-sensitive A2780 cells (Fig. 1D, right panel), but not in its isogenic but platinum-resistant counterpart A2780cis (Supplementary Fig. S1E). Conversely, overexpression of FLYWCH1 (Fig. 1E and Supplementary Fig. S1F) induced a mild sensitization of A2780 cells to cPt treatment (Fig. 1F and Supplementary Fig. S1G), while having no effect on the survival of A2780cis cells (Supplementary Fig. S1G and H). Since FLYWCH1 expression decreased upon early resistance development (Fig. 1C), we aimed to assess whether there are also changes in FLYWCH1 expression in long-term resistant cells (Supplementary Fig. S1B). Thus, we compared FLYWCH1 expression between the platinum-sensitive A2780 and resistant A2780cis cells and discovered that FLYWCH1 expression is significantly decreased in resistant cells (Fig. 1G). Further, we used curated data from the Cancer Cell Line Encyclopaedia (CCLE) [70] with known IC_50_ value and grouped them into platinum-sensitive (IC_50_< 20 μM) and -resistant (IC_50_> 20 μM) cell lines [16]. We noticed that resistant cell lines have a trend of lower FLYWCH1 expression levels compared to sensitive cells (Supplementary Fig. S2A). In summary, FLYWCH1 is a modulator of platinum resistance in EOC cell lines, and the loss of FLYWCH1 promotes resistance development, whereas a gain in FLYWCH1 expression leads to further sensitization in A2780 cells. Furthermore, both early and long-term resistance are linked to reduced FLYWCH1 expression.

### Low FLYWCH1 expression is associated with a poor prognosis for patients with EOC

Next, we were interested in determining the role of FLYWCH1 in a clinical context and investigated FLYWCH1 expression in three PDO lines of donors with different progression-free survival (PFS) times and clinical outcomes (long PFS, recurrence, death) (Fig. 1H and Supplementary Table S3). Interestingly, PDOs from patients with recurrence or death showed a decreased expression of FLYWCH1 compared to the patient with long PFS (Fig. 1I). The extent of decrease in FLYWCH1 expression was comparable with the reduction in sensitive versus resistant cell lines (Fig. 1G). To determine whether FLYWCH1 has an effect on overall survival (OS) of patients with EOC, we analysed a clinically well characterized cohort of 155 chemotherapy-naive patients with primary EOC [43, 44, 71] by immunohistochemistry (IHC). We stained patient tissue samples for FLYWCH1 (Fig. 1J) and correlated immunoreactive scores (IRSs) as a measure for FLYWCH1 expression with OS. Low expression of FLYWCH1 was correlated with a significant decrease in median OS from 125 months in patients with high FLYWCH1 expression to 78 months for patients with low FLYWCH1 expression (Fig. 1K). In line with this, publicly available data from the TCGA Research Network (https://www.cancer.gov/tcga) for ovarian cancer demonstrated that patients with FIGO III or IV ovarian cancer display a trend of decreased FLYWCH1 expression compared to patients with FIGO II tumours (Supplementary Fig. S2B). Additionally, patients that developed recurrence after initial treatment and patients with stable or progressive disease showed reduced FLYWCH1 expression compared to patients without recurrence or patients with complete remission (Supplementary Fig. S2C and D). Altogether these findings imply a role of FLYWCH1 in platinum-resistance development and propose FLYWCH1 as a potential prognostic marker for the response of EOC patients to platinum-based chemotherapy.

### Resistance development leads to loss in colocalization of H3K9me3 and FLYWCH1

Previous studies suggest an involvement of FLYWCH1 in Wnt signalling in both AML and CRC, where it was described to have an inhibitory role [19, 20]. Wnt signalling has been associated with drug resistance development in the past [12, 72, 73] and thus, we analysed the expression of Wnt genes in EOC cells with loss of FLYWCH1. However, we could not identify any trend that implies that FLYWCH1 is indeed an inhibitor of Wnt signalling in EOC (Supplementary Fig. S2E and F).

**Figure 2.**
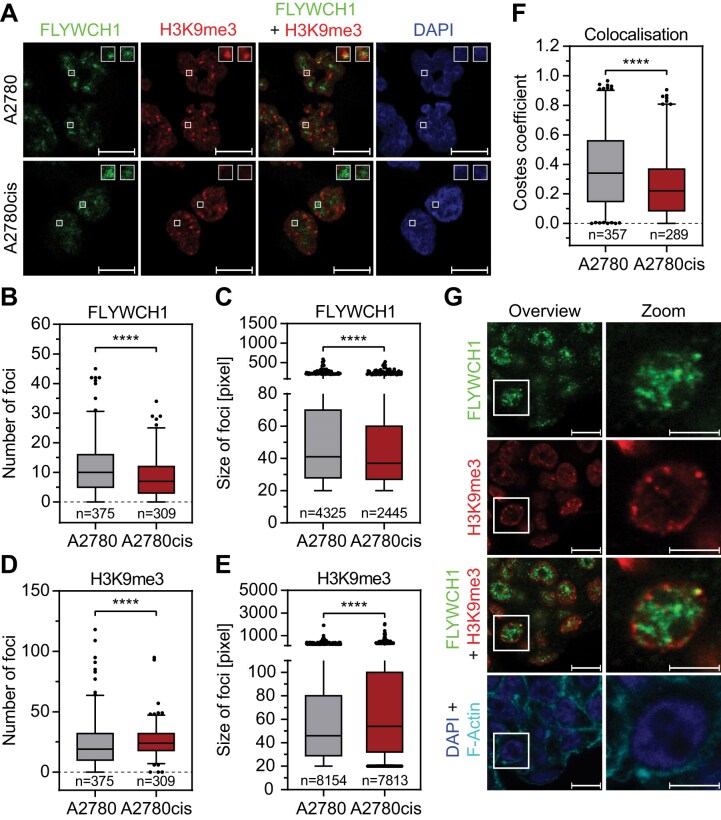
FLYWCH1 is associated with H3K9me3 and colocalization is lost during resistance acquisition. (**A**) Representative single focal plane images of immunofluorescence staining of FLYWCH1 and H3K9me3. Sensitive A2780 and resistant A2780cis cells were fixed and then stained with the indicated antibodies. Insets show enlarged images of the indicated foci; scale bar: 10 μm. (**B–** **E**) Quantification of maximum-intensity-projections of immunostaining images in panel (A) using CellProfiler. Analysis was performed on the indicated number of nuclei (B and D) or foci (C and E). Box plot with 2.5 and 97.5 percentiles, *n* = 3, statistical analysis: Mann–Whitney U test (****P ≤ 0.0001). (B and C) Quantification of number (B) and area (C) of FLYWCH1 foci. (D and E) Quantification of number (D) and area (E) of H3K9me3 foci. (**F**) Quantification of colocalization of FLYWCH1 and H3K9me3 from single focal plane immunofluorescence images in panel (A) using the Costes correlation coefficient. Analysis was performed on the indicated number of nuclei. Box plot with 2.5 and 97.5 percentiles, *n* = 3, statistical analysis: Mann–Whitney *U*-test (****P ≤ 0.0001). (**G**) Representative single focal plane images of immunofluorescence staining of FLYWCH1 and H3K9me3 in an EOC PDO with high FLYWCH1 expression. PDOs were fixed and then stained with the indicated antibodies. Scale bar: 10 μm, scale bar (zoom): 5 μm.

Recent proximity biotinylation experiments suggest an association of FLYWCH1 with H3K9me3 and ChIP-seq for FLYWCH1 and H3K9me3 showed a genome wide overlap in HeLa cells [22]. To analyse if FLYWCH1 and H3K9me3 also colocalize in EOC cells, we performed immunostainings for FLYWCH1 and H3K9me3 in sensitive A2780 and resistant A2780cis cells (Fig. 2A and Supplementary Fig. S3A). We discovered a significant reduction in the number, area and intensity of FLYWCH1 foci in resistant A2780cis cells (Fig. 2B and C, and Supplementary Fig. S3B), as already implied by the qPCR data (Fig. 1G). On the other hand, the H3K9me3 foci showed the opposite effect and increased in number, size and intensity in resistant cells (Fig. 2D and E, and Supplementary Fig. S3C). The distinct colocalization of FLYWCH1 and H3K9me3 in sensitive A2780 cells significantly decreased in resistant A2780cis cells (Fig. 2A and F). While the resistant cells still displayed some FLYWCH1 foci, these did not colocalize with H3K9me3, whereas most FLYWCH1 foci in the sensitive A2780 cells colocalized with H3K9me3 (Supplementary Fig. S3D). We validated this by transfecting A2780 cells with FLYWCH1 and subsequent antibody-based detection of H3K9me3 and observed a striking colocalization of the ectopically expressed FLYWCH1 and H3K9me3 (Supplementary Fig. S3E). This colocalization was also observed in PDOs (Fig. 2G), but decreased in PDOs from patients with recurrence or death compared to the patients with long PFS (Supplementary Fig. S4A and B). Interestingly, we also observed a distinct colocalization of FLYWCH1 and H3K27me2/me3 in EOC cells (Supplementary Fig. S3F). This is in line with our finding that EZH2, which is part of the Polycomb repressive complex 2 (PRC2) that establishes the repressive histone mark H3K27me3 [74], was also one of the significantly enriched hits in our screen (Fig. 1A). To further validate the interaction of FLYWCH1 and H3K9me3, we transiently coexpressed FLYWCH1 together with CBX3 (HP1γ), a well-known H3K9me3 binding protein [75], and observed a distinct colocalization of FLYWCH1 with CBX3 (Supplementary Fig. S5A). In summary, FLYWCH1 colocalizes with H3K9me3, H3K27me3, and well-known corepressors, such as CBX3, in distinct chromatin sub-compartments (CSCs) but the colocalization between FLYWCH1 and H3K9me3 is lost in resistant cells.

### FLYWCH1 and H3K9me3 colocalize in distinct chromatin sub-compartments

Since previous studies have shown that FLYWCH1 does not directly bind to H3K9me3 [22], we hypothesized that there is another factor mediating the localization of FLYWCH1 to this modification. The AlphaFold structure prediction of FLYWCH1 shows five known FLYWCH1-type zinc finger domains predicted with very high confidence, while the rest of the protein is mainly predicted to be disordered regions (Supplementary Fig. S5B) [76, 77]. Thus, we employed the Predictor of Natural Disordered Regions (PONDR, http://www.pondr.com) [78] to identify potential IDRs, which confirmed the AlphaFold predictions (Supplementary Fig. S5C). IDRs within proteins can contribute to the formation of biomolecular condensates, aiding the formation of specific CSCs [79–83]. Several chromatin-associated cofactors, including members of the HP1 family, localize to such CSCs by forming biomolecular condensates in the nucleus [84]. CBX3 can interact with the DNA damage compartment through the formation of nuclear condensates and this condensate formation is enhanced in the presence of certain interacting proteins [85]. Based on the observed colocalization of FLYWCH1 with CBX3 and the presence of multiple IDRs, we hypothesized that FLYWCH1 might be incorporated into nuclear biomolecular condensates containing H3K9me3. As an initial test we treated A2780 cells with increasing concentrations of 1,6-hexanediol and subsequently stained the cells for FLYWCH1 and H3K9me3 (Supplementary Fig. S5D). Treatment with 1,6-hexanediol is widely used as an indicator for biomolecular condensates, while also being debated for other side effects [86]. Indeed, treatment with 1,6-hexanediol induced a complete loss in FLYWCH1 foci (Supplementary Fig. S5E), a significant reduction in H3K9me3 foci (Supplementary Fig. S5F) and a reduction in colocalization of FLYWCH1 and H3K9me3 (Supplementary Fig. S5G). These data imply that the localization of FLYWCH1 to H3K9me3 is at least in part driven by the formation of nuclear condensates.

To analyse whether FLYWCH1 can indeed form biomolecular condensates *invitro*, we fused recombinant full-length CBX3 to dsRed-monomer [87] and the second IDR of FLYWCH1 (Supplementary Fig. S5C) to mVenus. We focused our analysis on the N- and C-terminal IDRs of FLYWCH1 to reduce potential risks of protein misfolding and aggregation. To investigate droplet formation, we incubated CBX3 or FLYWCH1-IDR2 in combination with each other or fluorophore alone in the presence of a crowding buffer. While the fluorophores alone did not lead to any droplet formation, incubation of either CBX3 or FLYWCH1-IDR2 in the crowding buffer led to the formation of distinct droplets (Supplementary Fig. S5H). When FLYWCH1-IDR2 and CBX3 were incubated together, we observed a global overlap of FLYWCH1-IDR2 and CBX3 droplets (Supplementary Fig. S5H), indicating that FLYWCH1 and CBX3 might colocalize because they are part of the same nuclear condensates. To investigate whether IDR1 and IDR2 of FLYWCH1 (Supplementary Fig. S5C) alone are sufficient for its association with CBX3, we ectopically coexpressed IDR1 or IDR2 in combination with CBX3 in A2780 cells. This showed that the IDR alone is not sufficient for the localization of FLYWCH1 to the CBX3 CSC in cells (Supplementary Fig. S5I). This is most likely caused by the absence of multivalent interactions or a missing contribution from the zinc fingers of FLYWCH1 to find their target. Taken together these data suggest that while IDR2 of FLYWCH1 has the ability to form nuclear condensates *invitro* and FLYWCH1 is localized to the same CSCs as CBX3 in cells, the IDRs of FLYWCH1 alone are not sufficient to induce foci formation within the nucleus. This indicates that the ability of FLYWCH1 to form nuclear condensates is only in part responsible for the colocalization with H3K9me3 and a potential interaction with other partners and DNA through the zinc finger domains of FLYWCH1 might play an important role in this process.

### Cisplatin treatment leads to an increase in FLYWCH1 expression and H3K9me3 levels

**Figure 3.**
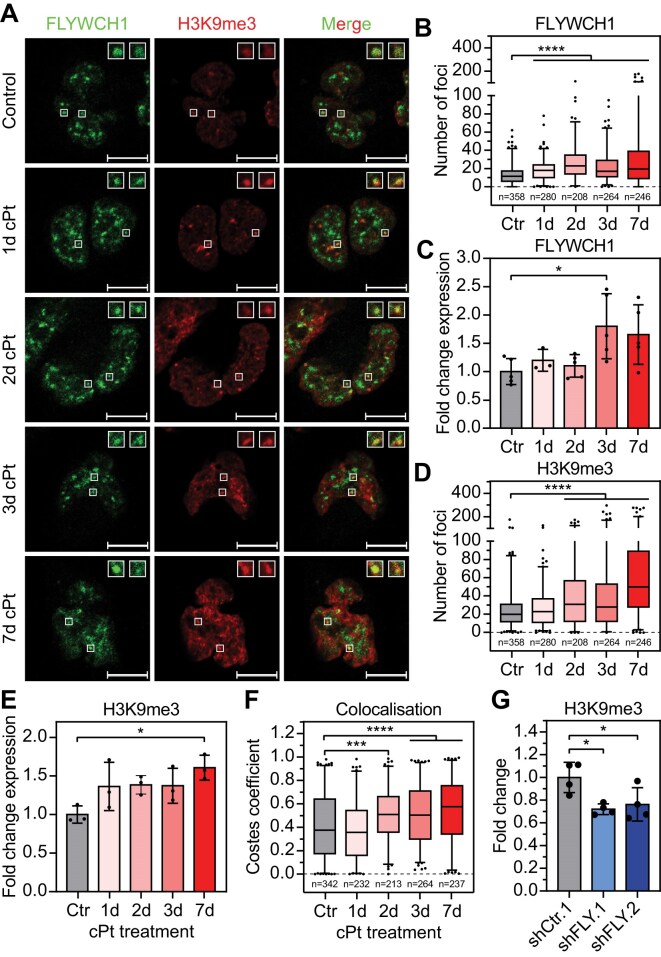
FLYWCH1 expression is correlated with H3K9me3 levels. (**A**) Representative single focal plane images of immunofluorescence staining of FLYWCH1 and H3K9me3. Sensitive A2780 were treated with 1 μM cPt for the indicated time points. Cells were fixed and then stained with the indicated antibodies. Insets show enlarged images of the indicated foci. Scale bar: 10 μm. (**B** and **D**) Quantification of maximum-intensity-projections of immunostaining images in panel (A) using CellProfiler. Number of FLYWCH1 (B) and H3K9me3 (D) foci was analysed in the indicated number of nuclei. Box plot with 2.5 and 97.5 percentiles, *n* = 3, statistical analysis: Kruskal–Wallis test and Dunn’s multiple comparisons test (****P ≤ 0.0001). (**C**) Gene expression of FLYWCH1 (RT-qPCR) in A2780 cells following cPt treatment. Cells were treated with 1 μM cPt for the indicated time points and FLYWCH1 expression levels determined relative to SDHA control and untreated cells (Ctr). Mean ± SD, *n* = 3–5, statistical analysis: ordinary one-way ANOVA and Dunnett’s multiple comparisons test (*P ≤ 0.05). (**E**) H3K9me3 level in A2780 cells following cPt treatment. Cells were treated with 1 μM cPt for the indicated time points, fixed and stained using an antibody against H3K9me3. H3K9me3 signal was background corrected and is shown relative to untreated cells (Ctr). Mean ± SD, *n* = 3, statistical analysis: ordinary one-way ANOVA and Dunnett’s multiple comparisons test (*P ≤ 0.05). (**F**) Quantification of colocalization of FLYWCH1 and H3K9me3 from single focal plane immunofluorescence images in panel (A) using the Costes correlation coefficient. Analysis was performed on the indicated number of nuclei. Box plot with 2.5 and 97.5 percentiles, *n* = 3, statistical analysis: Kruskal–Wallis test and Dunn’s multiple comparisons test (***P ≤ 0.001, ****P ≤ 0.0001). (**G**) H3K9me3 level in A2780 cells following FLYWCH1 KD. KD was induced by expression of two independent shRNA targeting FLYWCH1 (shFLY) or control shRNA for 7 weeks. Cells were then fixed and stained using an antibody against H3K9me3. H3K9me3 signal was background and batch corrected and is shown relative to shCtr cells. Mean ± SD, *n* = 4, statistical analysis: ordinary one-way ANOVA and Dunnett’s multiple comparisons test (*P ≤ 0.05).

Given that FLYWCH1 and H3K9me3 colocalize in platinum-sensitive cells but that this interaction is lost in long-term resistant cells, we next aimed to investigate the effect of acute cPt treatment on FLYWCH1 and its association with H3K9me3. To this end, we treated A2780 cells with a GI_25_ dose of cPt and performed immunostaining for FLYWCH1 and H3K9me3 (Fig. 3A and Supplementary Fig. S6A). We found that acute cPt treatment significantly increased the number of FLYWCH1 foci (Fig. 3B) with no changes in area (Supplementary Fig. S6B) but an increase in overall intensity (Supplementary Fig. S6C). We validated this observation by analysing the expression of FLYWCH1 following cPt treatment and discovered that acute cPt treatment led to a significant increase in FLYWCH1 expression (Fig. 3C), in conjunction with an increase in the foci number, area, and overall intensity of H3K9me3 (Fig. 3D, and Supplementary Fig. S6D and E). This was also reflected by the H3K9me3 levels, which significantly increase during acute cPt treatment (Fig. 3E). The observed increase in the number of FLYWCH1 and H3K9me3 foci was also accompanied by a significant increase in colocalization (Fig. 3F and Supplementary Fig. S6F). The identified correlation of FLYWCH1 and H3K9me3 levels under acute treatment was also observed in sensitive A2780 cells with FLYWCH1 KD, which showed a significant decrease in H3K9me3 levels (Fig. 3G). Next, we ectopically expressed FLYWCH1 and CBX3 in combination with vehicle or cPt treatment. However, we identified no notable differences, suggesting that cPt treatment does not interfere with the colocalization ability of FLYWCH1 and H3K9me3 itself (Supplementary Fig. S6G), but rather the increase in colocalization is caused by an increase in protein availability. Altogether these data show that FLYWCH1 and H3K9m3 levels correlate in sensitive A2780 cells, and acute treatment leads to an increase in both FLYWCH1 and H3K9me3.

### FLYWCH1 is not associated with acute DNA damage response upon cPt treatment

Platinum treatment induces DNA damage in cells and the resulting activation of the DNA damage response leads to the accumulation of DNA double strand breaks [88]. During the DNA damage response, double strand breaks are marked with H3K9me3, thereby stimulating the repair process [89]. Thus, the observed increase in both FLYWCH1 and H3K9me3 could point to a potential involvement of FLYWCH1 in the DNA damage repair process, as proposed recently [21]. To investigate this further, we performed immunostainings for FLYWCH1 and γH2A.X, a well-established marker for DNA double strand breaks, in A2780 cells following cPt treatment (Supplementary Fig. S7A). Treatment with cPt led to the already observed increase in the number of FLYWCH1 foci upon treatment and reduction in resistant cells (Supplementary Fig. S7B). Again, we demonstrated only minor changes in the area of FLYWCH1 foci upon cPt treatment and a significant decrease in foci size in A2780cis cells (Supplementary Fig. S7C). As expected, treatment with cPt induced an activation of the DNA damage response as evident from the significant increase in both number and size of γH2A.X foci (Supplementary Fig. S7D and E). We observed the strongest increase in the number of γH2A.X foci after 2 days of treatment, which then slowly declined. Resistant A2780cis cells showed a reduced DNA damage response compared to treated sensitive cells, while still showing a significant increase compared to untreated sensitive cells (Supplementary Fig. S7D). While the γH2A.X signal in untreated A2780 cells was rather weak, most FLYWCH1 foci seemed to colocalize with γH2A.X (Supplementary Fig. S7F and G). Surprisingly, cPt treatment significantly reduced the overall colocalization of FLYWCH1 with γH2A.X, both in treated sensitive and resistant cells (Supplementary Fig. S7F). During treatment, FLYWCH1 foci were still found in regions with weak γH2A.X signal; however, the strong γH2A.X foci that appeared upon cPt treatment did not colocalize with FLYWCH1 (Supplementary Fig. S7G).

This implies that while FLYWCH1 is localized to sites of endogenous DNA damage repair, it does not seem to be involved in the DNA damage response in EOC cells evoked by exogenous stimuli such as platinum treatment.

### FLYWCH1 modulates the expression of genes linked to pathways implicated in platinum resistance

**Figure 4.**
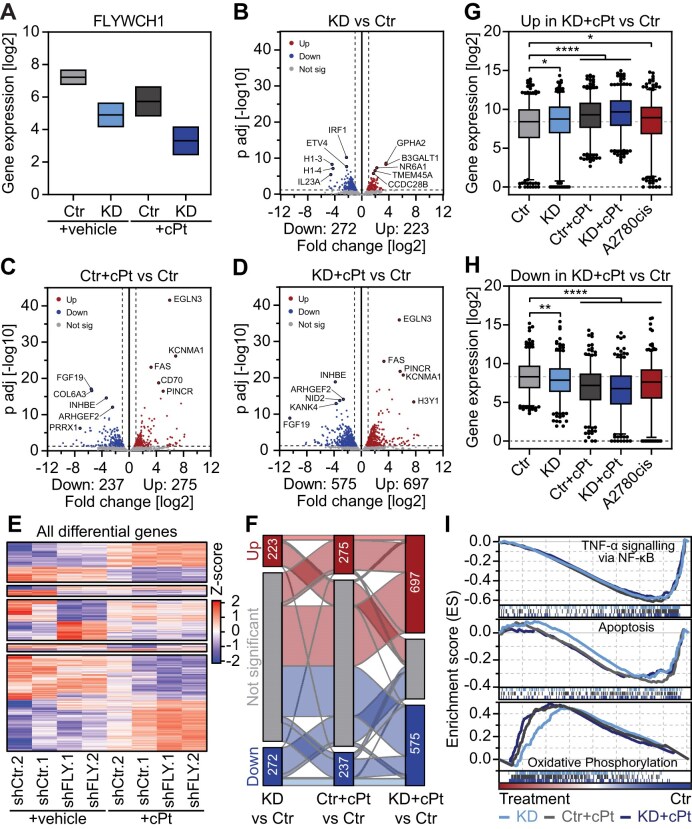
FLYWCH1 regulates genes associated with resistance development. (**A**) Analysis of FLYWCH1 expression from RNA-seq data of vehicle and cPt treated A2780 cells with Ctr or FLYWCH1 KD. RNA-seq was performed on cells expressing two independent hairpins (shCtr or shFLY) for 7 weeks and were treated with vehicle or 1 μM cPt for 6 weeks. Floating bars with min to max and line at mean, *n* = 2. (**B**–**D**) Volcano plot showing gene expression changes in cells following FLYWCH1 KD (B), early platinum-resistance development (C), and cells that gained resistance with the aid of FLYWCH1 loss (D) relative to untreated control cells. RNA-seq was performed on cells expressing two independent hairpins (shCtr or shFLY) for 7 weeks and were treated with vehicle or 1 μM cPt for 6 weeks. Statistical significance was determined using DEseq2. Significantly up- or downregulated genes are indicated by colour and their number is indicated below. (**E**) Differentially regulated genes from panel (B–D) were grouped based on their differential expression and each group clustered using k-means clustering. (**F**) Alluvial plot showing transitions between differentially expressed genes in panels (B–D). Genes that show up- or downregulation in all three conditions are highlighted in light red (up) and light blue (down). (**G** and **H**) Average expression of all 575 upregulated (H) and 697 downregulated (I) genes in resistance development with the aid of FLYWCH1 loss (KD+cPt). Normalized count was averaged over the two independent shRNAs/repeats and plotted for each condition. Box plot with 2.5 and 97.5 percentiles, statistical analysis: Kruskal–Wallis test and Dunn’s multiple comparisons test (*P ≤ 0.05, **P ≤ 0.01, ****P ≤ 0.0001). (**I**) GSEA was performed for genes of the most enriched and depleted Hallmark pathways in FLYWCH1 KD as well as for all Hallmark apoptosis genes. Enrichment scores are shown for all treatment conditions (KD, Ctr+cPt and KD+cPt versus Ctr) and colour coded.

To further understand how FLYWCH1 affects the response to cPt in EOC cells and given that it contains five zinc finger domains and is associated with H3K9me3, we aimed to determine transcriptome alterations connected to FLYWCH1 loss. To this end we performed RNA-seq in cells expressing shRNAs targeting FLYWCH1 (KD/shFLY) and compared gene expression to cells expressing control shRNAs (Ctr/shCtr). In addition, we analysed gene expression in early resistant cells (Supplementary Fig. S1B) that have developed resistance without (Ctr+cPt/shCtr+cPt) or with the aid of FLYWCH1 KD (KD+cPt/shFLY+cPt) and compared it to untreated cells (Fig. 4A and Supplementary Fig. S8A). FLYWCH1 suppression (KD) led to the significant upregulation of 223 and downregulation of 272 genes (Fig. 4B, Supplementary Fig. S8G and H, and Supplementary Table S4). Early resistance development independent of FLYWCH1 (Ctr+cPt) led to a similar number of dysregulated genes, but more pronounced changes (Fig. 4C, Supplementary Fig. S8G and H, and Supplementary Table S5). Interestingly, we observed the strongest effect on gene expression upon resistance development in combination with FLYWCH1 KD (KD+cPt), where 697 genes were upregulated and 575 genes downregulated (Fig. 4D, Supplementary Fig. S8G and H, and Supplementary Table S6). Next, we identified different groups of DEGs (Fig. 4E and Supplementary Fig. S8B–F), among them a group of genes connected to early resistance development independent of FLYWCH1 KD (Supplementary Fig. S8B) and a group of genes differentially regulated in both FLYWCH1 KD and early resistance development (Supplementary Fig. S8C). Additionally, we also discovered genes that only showed significant changes in expression in one of the conditions (Supplementary Fig. S8D–F). Overall, the biggest differences in gene expression were caused by resistance development under FLYWCH1 KD (KD+cPt) (Supplementary Fig. S8D), followed by the effect of resistance development alone (Ctr+cPt) (Supplementary Fig. S8E) and FLYWCH1 suppression alone (KD) (Supplementary Fig. S8F). We identified 79 genes that are significantly downregulated and 52 genes that are significantly upregulated in both FLYWCH1 KD and upon early resistance development with FLYWCH1 KD (KD+cPt) (Supplementary Fig. S8C, G, and H). Surprisingly, this is the minority of the dysregulated genes and most genes that changed their expression in FLYWCH1 KD showed no significant changes upon early resistance development (Fig. 4F). Thus, we had a closer look at the expression of the genes in these groups. While genes that increase in expression upon FLYWCH1 KD showed no significant changes in any of the other conditions (Supplementary Fig. S8I), genes that are downregulated upon FLYWCH1 KD are also significantly downregulated in early resistant development (Ctr+cPt) and even stronger in early resistance development with FLYWCH1 KD (KD+cPt) (Supplementary Fig. S8J). Interestingly, genes that are differentially expressed upon early resistance development with or without suppression of FLYWCH1 (Ctr+cPt or KD+cPt) showed similar changes in FLYWCH1 KD without treatment (KD) and in long-term resistant A2780cis cells (Supplementary Fig. S1B), albeit to a lesser extent (Fig. 4G and H, and Supplementary Fig. S8K and L). This implies that FLYWCH1 KD aids in resistance development by accelerating the deregulation of genes important for resistance development against platinum-based chemotherapy.

Next, we performed gene set enrichment analysis (GSEA) to identify HALLMARK pathways linked with the differential clusters (Fig. 4E, Supplementary Fig. S9A–D, and Supplementary Table S7). Surprisingly, early resistance development (Ctr+cPt) led to a downregulation of genes connected to various signalling pathways, such as inflammatory signalling via tumor necrosis factor α (TNFα)/nuclear factor 'kappa-light-chain-enhancer' of activated B-cells (NF-κB) and janus kinase (JAK)/signal transducers and activators of transcription (STAT), but also kirsten rat sarcoma virus (KRAS), transforming growth factor β (TGF-β) and Wnt signalling as well as genes linked to EMT and apoptosis (Supplementary Fig. S9A). On the other hand, early resistance development led to the upregulation of only two pathways, oxidative phosphorylation and Myc target genes (Supplementary Fig. S9A). Interestingly, we found the deregulation of the same cellular pathways also in FLYWCH1 KD and early resistance development with the aid of FLYWCH1 KD (KD+cPt) (Fig. 4I and Supplementary Fig. S9B–D). Recent studies showed that oxidative phosphorylation is involved in general resistance development, as it is frequently upregulated in resistant cancer cells and treatment with oxidative phosphorylation inhibitors can resensitize resistant cancer cells to treatment [90, 91]. This implies that loss of FLYWCH1 aids the resistance development process by deregulating pathways important for the acquisition of early platinum resistance. On the one hand, loss of FLYWCH1 leads to downregulation of apoptosis related genes and inflammatory pathways, while on the other hand it upregulates the expression of Myc target genes and genes linked to oxidative phosphorylation, thus driving the resistant phenotype. Unexpectedly, re-expression of FLYWCH1 in A2780cis cells (Supplementary Fig. S10A) was ineffective and did not lead to significant changes in gene expression (Supplementary Fig. S10B and C, and Supplementary Table S8).

Taken together, these data suggest that loss of FLYWCH1 accelerates platinum-resistance by deregulating genes involved in the resistance development process; however, FLYWCH1 is ineffective in re-establishing the sensitive state.

### FLYWCH1 induces gene silencing in a cellular reporter system

**Figure 5.**
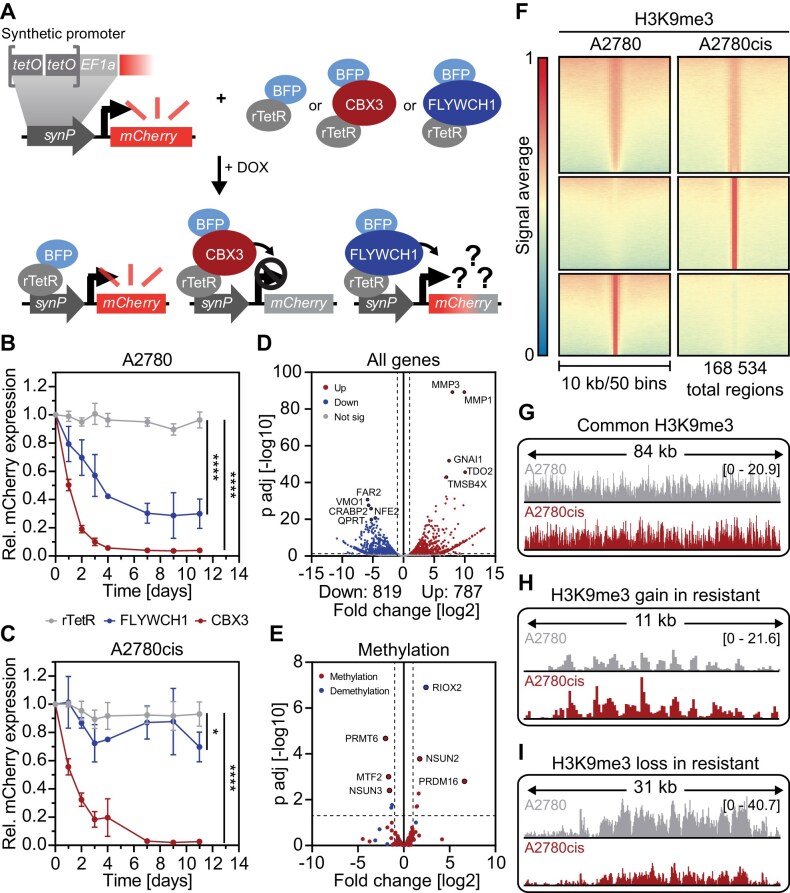
Resistance development is accompanied by changes in FLYWCH1 function and alterations in H3K9me3 at defined genomic loci. (**A**) Schematic representation of the fluorescent reporter assay. Cells are transduced with a fluorescent reporter, in which mCherry is expressed from a synthetic promoter containing multiple tetO-binding sites. Cells are additionally transduced to express an effector (CBX3 or FLYWCH1) coupled to rTetR and BFP. Dox addition leads to recruitment of the effectors to the synthetic promoter, where the effector can exert its gene regulatory effect. Figure modified from [40]. (**B** and **C**) Reporter assay in A2780 (B) and A2780cis (C) cells expressing different effectors: FLYWCH1, the positive control CBX3 and the negative control rTetR. Shown is mCherry expression of effector+/BFP+ cells relative to noDox and the initial measurement. Mean ± SD, *n* = 3, statistical analysis: two-way ANOVA and Dunnett’s multiple comparisons test (*P ≤ 0.05, ****P ≤ 0.0001). (**D** and **E**) Volcano plot showing changes in gene expression between resistant A2780cis and sensitive A2780 cells. Panel (D) shows all genes, and panel (E) shows only genes associated with methylation or demethylation via its GO terms. Statistical significance was determined using DEseq2. Genes significantly up- or downregulated in A2780cis cells are indicated by colour and their number is indicated below. (**F**) Heatmaps showing the average H3K9me3 ChIP-seq signal for A2780 and A2780cis cells, sorted by the highest average signal in A2780 cells and plotted with a 10 kb window in 50 bins around the peak centre. Regions with differential H3K9me3 signal were identified by K-means clustering. (**G–** **I**) Representative ChIP-seq tracks of H3K9me3 intensity in A2780 and A2780cis for clusters identified in panel (F).

Next, we set out to investigate how FLYWCH1 exerts its gene-regulatory effect and deployed a previously published reporter assay that was developed in our lab [40]. In brief, A2780 and A2780cis cells were transduced with a reporter cassette expressing mCherry from a synthetic promoter harbouring multiple tetO-binding sites. Additionally, cells were transduced with FLYWCH1 fused to the reverse Tet repressor (rTetR) and BFP. Upon doxycycline (dox) addition to the cells, rTetR-FLYWCH1 is recruited to the synthetic promoter via rTetR and can exert its gene-regulatory effect (Fig. 5A). We compared the effects of rTetR-FLYWCH1 to the positive control rTetR-CBX3, which leads to the deposition of H3K9me3 and thereby silencing of the mCherry reporter gene, and the negative control rTetR alone, which has no effect on reporter expression (Fig. 5B and C). Recruitment of FLYWCH1 to the synthetic promoter in sensitive A2780 cells led to significant silencing of the reporter gene down to 40% (Fig. 5B). In line with RNA-seq data of A2780cis cells with FLYWCH1 reintroduction (Supplementary Fig. S10), FLYWCH1 recruitment to the reporter in A2780cis cells only had little effects on gene expression (Fig. 5C), suggesting that FLYWCH1 does not exert its repressive role alone but with the aid of other coregulators or coregulator complexes that might be downregulated in resistant cells.

Next, we analysed gene expression changes in sensitive A2780 and resistant A2780cis cells and discovered 819 downregulated and 787 upregulated genes in A2780cis cells (Fig. 5D and Supplementary Table S9). These were connected to a downregulation of Notch signalling and an upregulation of the interferon response, Myc target genes and EMT (Supplementary Fig. S11A and Supplementary Table S10). The genes that were upregulated in A2780cis cells showed no significant changes in FLYWCH1 KD or early resistance development (Supplementary Fig. S11B), whereas the genes downregulated in long-term resistant cells also showed downregulation in early resistance development, albeit to a lesser extent (Supplementary Fig. S11C). To identify putative cofactors of FLYWCH1 that are lost in long-term resistant cells and could explain the lack of silencing efficiency of FLYWCH1, we filtered our RNA-seq data for epigenetic cofactors and categorized the genes depending on their function (Supplementary Fig. S11D and E). Since our data and previous publications [22] showed a link between FLYWCH1 and H3K9me3, we first focused our analysis on epigenetic factors connected to methylation and demethylation. The ribosomal oxygenase 2 (RIOX2/MINA) was significantly upregulated in resistant cells (Fig. 5E) and it was previously reported to be involved in the demethylation of H3K9me3 [92]. Moreover, we discovered a significant downregulation of the protein arginine methyltransferase 6 (PRMT6) and the metal response element binding transcription factor 2 (MTF2) in resistant cells (Fig. 5E). PRMT6 has been described to asymmetrically methylate arginine 17 on histone 3 (H3R17me2a), which is connected to active transcription [93]. In contrast, PRMT6 also induces asymmetric di-methylation of histone H3 arginine 2 (H3R2me2a), which was shown to inhibit H3K4me3, thus making H3R2me2a a repressive mark [94, 95]. Similarly, MTF2 is also associated with repressive chromatin and leads to gene silencing via recruitment of PRC2 and subsequent H3K27me3 deposition [96]. In addition to epigenetic factors associated with methylation, we also identified changes in expression of transcriptional regulators and putative FLYWCH1 coregulators in long-term resistant cells (Supplementary Fig. S11F and G), which could explain why re-introduction of FLYWCH1 does not lead to changes in gene expression in resistant A2780cis cells.

In summary, these data show that FLYWCH1 can induce gene silencing in A2780 cells but is ineffective in doing so in long-term resistant cells. This suggest that the gene-silencing effect of FLYWCH1 depends on other coregulators that are downregulated in long-term resistant cells.

### FLYWCH1 knockdown and resistance development induce changes in H3K9me3 at distinct genomic loci

Next, we aimed to investigate whether the observed reduction of FLYWCH1 in A2780cis cells is also accompanied with changes in H3K9me3. To this end we performed ChIP-seq for H3K9me3 in sensitive A2780 and resistant A2780cis cells and identified regions that gain or lose H3K9me3 signal in resistant cells (Fig. 5F–I and Supplementary Fig. S12A–C). Interestingly, we identified almost as many up- and downregulated regions in A2780cis cells as regions with similar H3K9me3 signal (common regions) (Supplementary Fig. S12D). We discovered that while the common regions have an average size of 1000 bp, the differential regions are much smaller with only 380 bp for regions that gain and 440 bp for regions that lose H3K9me3 in resistant cells (Supplementary Fig. S12E). This indicates that the common and differential regions might be associated with different types of repeat elements, as different repeat classes were shown to exhibit different sizes, but are relatively small and decorated with H3K9me3 [97].

**Figure 6.**
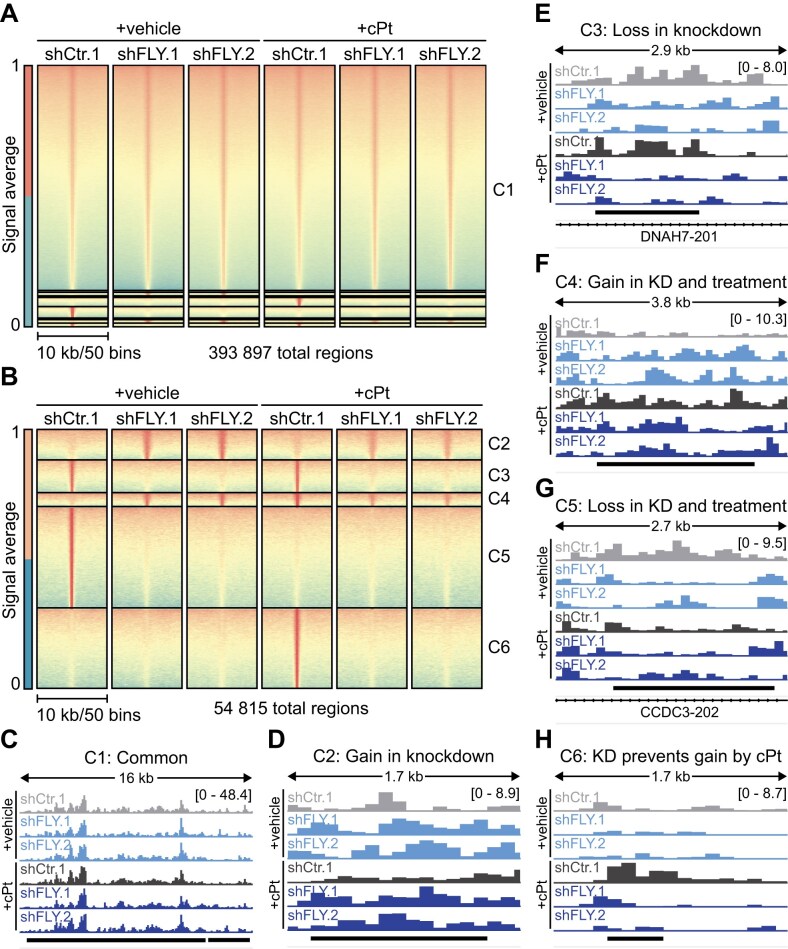
FLYWCH1 KD and early resistance development are associated with changes in H3K9me3 at distinct genomic loci. (**A** and **B**) Heatmaps showing the average H3K9me3 ChIP-seq signal of control and FLYWCH1 KD cells, as well as cells that developed platinum resistance dependent or independent of FLYWCH1 loss. ChIP-seq was performed on cells expression two independent hairpins (shCtr or shFLY) for 7 weeks and were treated with vehicle or 1 μM cPt for 6 weeks. Signals are sorted by the highest average signal in untreated shCtr cells and plotted with a 10 kb window in 50 bins around the peak centre. Regions with differential H3K9me3 signal were identified by K-means clustering. B shows zoom-in of relevant clusters used in downstream analyses. (**C–** **H**) Representative ChIP-seq tracks of H3K9me3 intensity for the data shown in A and B.

Having discovered changes in H3K9me3 in sensitive and resistant cells, we aimed to assess whether FLYWCH1 KD and early resistance development also induce changes in H3K9me3. To this end we performed H3K9me3 ChIP-seq in cells expressing shRNAs targeting FLYWCH1 or control shRNAs. Additionally, cells were either left untreated or treated with cPt until they developed platinum resistance without or with the aid of FLYWCH1 loss (Fig. 6A–H and Supplementary Fig. S13A–F). The number of differential H3K9me3 regions of FLYWCH1 KD and early resistance development was much lower compared to the number of differential regions of long-term resistant cells (Figs 5F, 6A, and B), but we still identified a considerable number of regions with differential signal (Fig. 6B and Supplementary Fig. S13G). We were most interested in clusters where FLYWCH1 KD influences H3K9me3 signal, either independent of resistance development (Loss or gain in KD) (Fig. 6D and E, and Supplementary Fig. S13A and B) or where FLYWCH1 KD leads to loss or gain in regions that also lose or gain H3K9me3 in early resistance development (Loss or gain in KD and cPt) (Fig. 6F and G, and Supplementary Fig. S13C and D). Additionally, we were also interested in the cluster where FLYWCH1 KD prevents a gain of H3K9me3 upon early resistance development (Fig. 6H and Supplementary Fig. S13E). These clusters are most helpful to understand the role that FLYWCH1 plays in the resistance development process. Interestingly, apart from the regions with similar H3K9me3 signal (common) (Fig. 6A and C, and Supplementary Fig. S13F), the three biggest clusters were all associated with loss of H3K9me3 in FLYWCH1 KD (Supplementary Fig. S13G), suggesting that FLYWCH1 not only interacts with H3K9me3 but can also influence H3K9me3 deposition, as we suspected already from our results with the cellular reporter system (Fig. 5B). Given that the differential regions in sensitive and resistant cells were on average less than half the size of the common regions, we next analysed the size distribution between the common and the most interesting differential clusters of FLYWCH1 KD (Supplementary Fig. S13H). We observed a similar effect in region size as we did for the differential regions of resistant cells (Supplementary Fig. S12E).

In summary, both loss of FLYWCH1 in resistant cells or by enforced suppression lead to changes in H3K9me3 at distinct genomic loci. Regions with differential H3K9me3 signal are smaller compared to the common regions, which raises the question as to whether the identified differential regions of FLYWCH1 KD, early resistance development and long-term resistance development might be connected to the same genomic elements.

### FLYWCH1 suppression leads to changes in H3K9me3 at repeat elements

**Figure 7.**
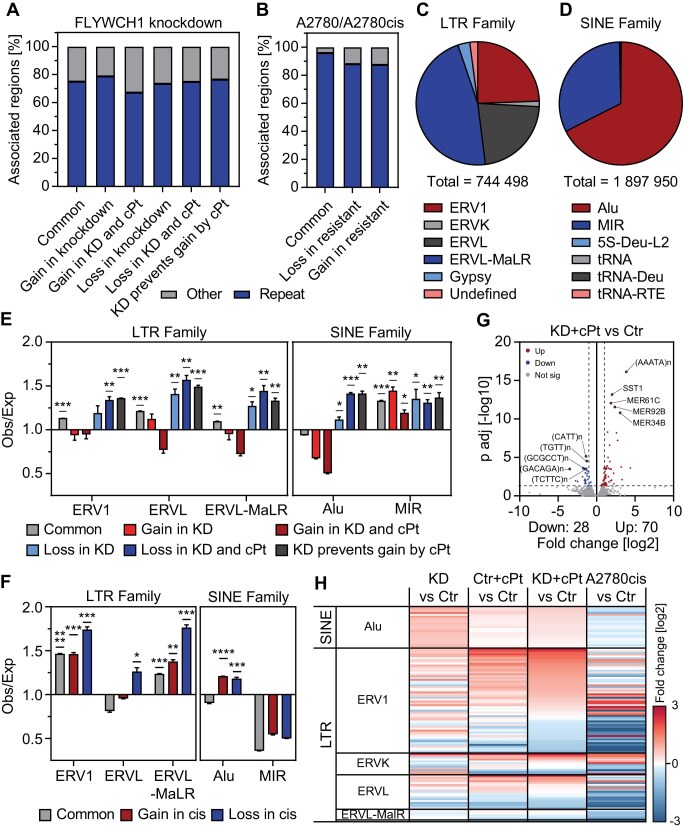
Repeat elements are associated with changes in H3K9me3 signal and expression upon resistance development and loss of FLYWCH1. (**A** and **B**) Percentage of common and differential H3K9me3 peaks from FLYWCH1 KD and resistance development (A) or A2780/A2780cis cells (B) overlapping with repeat elements. (**C** and **D**) Distribution of repeat elements in the subfamilies of the LTR (C) and SINE (D) class of repeats. (**E** and **F**) Observed/expected ratio of all common and differential regions from FLYWCH1 KD and resistance development (E) and A2780/A2780cis cells (F) with repeat elements of the three biggest LTR subfamilies (left) and two biggest SINE subfamilies (right). The observed incidence of the indicated repeat cluster was divided by the expected incidence if the peaks were distributed by random chance (shuffled bed file). Values higher than one indicate that regions are associated with the respective repeat more often than expected by random chance, values less than one indicate a depletion of those regions from the repeat. Bed files were randomly shuffled three times for *n* = 3 observed/expected ratios. Mean ± SD, statistical analysis: one sample *t*-test was performed to test if the mean is larger than a hypothetical mean of one (*P ≤ 0.05, **P ≤ 0.01, ***P ≤ 0.001, ****P ≤ 0.0001). (**G**) Volcano plot showing changes in repeat expression in cells that developed platinum-resistance with the aid of FLYWCH1 KD compared to untreated control cells. Statistical significance was determined using DEseq2. Significantly up- or downregulated repeats are indicated by colour and their number is indicated below. (**H**) Fold change of expression of repeats from the SINE and LTR family. Repeat elements were filtered and heatmap only shows elements with significant up- or downregulation in one of the conditions.

H3K9me3 is a mark mainly found in heterochromatin, where it ensures transcriptional silencing of repetitive DNA sequences but also genes [23]. While protein-coding genes only make up ∼3% of the human genome [98], >50% of the human genome is comprised of repeat elements [99]. When comparing the mean size of repeat elements from all repeat classes, we identified only two repeat classes that are on average >1 kb, namely the Satellite and Beta repeats (Supplementary Fig. S14A). All other repeat classes have an average size of <1 kb and most repeat classes are on average only 50–400 bp in size (Supplementary Fig. S14A and B). This is in line with the size of the differential regions observed in FLYWCH1 KD, early resistance development and long-term resistance development. Thus, we analysed how many of the common and differential H3K9me3 peaks from FLYWCH1 KD and early resistance development overlap with repeat elements and discovered that 67%–79% of the regions were associated with repeats elements (Fig. 7A). The association of H3K9me3 peaks with promoter regions was much lower and only 4%–12% of all peaks were found at promoter regions (Supplementary Fig. S14C). While 4%–6% of regions that lose H3K9me3 in FLYWCH1 KD and resistance development coincided with promoters, 10%–12% of regions that gain H3K9me3 were found at promoters (Supplementary Fig. S14C), implying that loss of FLYWCH1 might lead to a gain of H3K9me3 at promoter regions. Next, we analysed the differential H3K9me3 peaks between sensitive and resistant cells and noticed that 88%–96% of all regions in sensitive and resistant cells were associated with repeat elements (Fig. 7B). When looking at possible gene regulation by H3K9me3, we noticed that only ∼2% of H3K9me3 regions were located at gene promoters (Supplementary Fig. S14D). While the effects of FLYWCH1 KD on the H3K9me3 signal at promoters is higher than that of long-term resistance development, the number of differential H3K9me3 peaks found at promoters is very small, suggesting only minor effects on gene expression.

Altogether this data shows that FLYWCH1 KD, early and long-term resistance development lead to alterations of H3K9me3 at repeat elements.

### FLYWCH1 suppression alters H3K9me3 levels at distinct repeat element classes

Since most differential H3K9me3 regions coincided with repetitive regions, we performed an in-depth analysis of H3K9me3 changes at repeat elements. For this we determined enrichment (Obs/Exp > 1) or depletion (Obs/Exp < 1) of the common and differential H3K9me3 regions at different classes of repeat elements and discovered that the common regions were significantly enriched at almost all repeat types (Supplementary Fig. S14E). This is not surprising, as repetitive elements are well known to be decorated with H3K9me3 [23]. The differential H3K9me3 regions in FLYWCH1 KD and early resistance development on the other hand were only significantly enriched for LTR, retroposon, and SINE (Supplementary Fig. S14E), while the differential regions of sensitive and resistant cells only showed enrichment for beta, LTR, and retroposons (Supplementary Fig. S14F). We performed in depth analysis of H3K9me3 in the largest of these repeat classes, namely the SINE and LTR family (Supplementary Fig. S14G). Members of the LTR family are implicated with different diseases such as prostate cancer, breast cancer, or systemic lupus erythematosus and different types of ERVs are expressed depending on the cell type involved [100–102]. The LTR class has in total five subfamilies, but the majority of LTRs belong to the ERV1 (24.4%), ERVL (22.1%), or ERVL-MalR (46.8%) subfamilies (Fig. 7C). The SINE class has a total of six subfamilies, but most SINEs belong to the Alu (67.4%) and MIR (32.1%) subfamilies (Fig. 7D). Deregulation of Alu but not MIR elements is linked to many diseases including cancer [103–105]. Given that different repeat elements are connected to different diseases in different cell types, we focused our further analysis on the biggest subfamilies within the LTR and SINE classes of repeats to investigate if there are differences between the subfamilies. Members of the LTR subfamilies all showed similar trends in their enrichment to the overall effect observed in the parental LTR class (Fig. 7E and Supplementary Fig. S14E). All LTR subfamilies are significantly enriched for common regions, however the enrichment is increased in regions that lose H3K9me3 in FLYWCH1 KD and early resistance development (Fig. 7E). The analysis of the SINE class of repeats showed a different effect: while the Alu subfamily only showed significant enrichment in regions that lose H3K9me3 in FLYWCH1 KD, elements from the MIR subfamily are enriched in all types of regions to a similar extent (Fig. 7E). Next, we performed the same analysis on the differential regions of sensitive and resistant cells and again found that the enrichment among the LTR subfamilies is comparable to the overall enrichment in the parental LTR class (Fig. 7F and Supplementary Fig. S14F). While all subfamilies showed the highest enrichment for regions that lose H3K9me3 in resistant cells, elements from the ERV1 and ERV1-MalR subfamily also display enrichment in the common regions and regions that gain H3K9me3 in resistant cells (Fig. 7F). Interestingly, while overall the SINE class showed no significant enrichment (Supplementary Fig. S14F), repeat elements from the Alu subfamily were significantly enriched in regions with differential H3K9me3 signal in resistant cells (Fig. 7F). To validate our findings that FLYWCH1 suppression and resistance development both lead to loss of H3K9me3 in repeat elements, we analysed H3K9me3 signal at ERV1, ERVL, ERVL-MalR, Alu, and MIR elements. Again, we were able to identify clusters where H3K9me3 is lost upon FLYWCH1 KD and early resistance development (loss in KD and cPt) and regions where FLYWCH1 KD prevents an increase in H3K9me3 signal upon resistance development (KD prevents gain by cPt) (Supplementary Fig. S15A–G), validating our earlier findings (Fig. 7E). Interestingly, we noticed an overall reduction in H3K9me3 signal at these repeats in resistant A2780cis cells compared to the sensitive A2780 cells (Supplementary Fig. S15A and C–F).

Taken together, this data shows that FLYWCH1 KD and resistance development lead to deregulation of H3K9me3 at LTRs and Alu elements. While we discovered a general deregulation with both loss and gain in H3K9me3 in resistant A2780cis cells, FLYWCH1 KD alone mainly induces loss of H3K9me3 at LTR and Alu elements.

### Changes in H3K9me3 levels upon FLYWCH1 suppression influence expression of repeat elements

The profound changes in H3K9me3 signal at repeat elements prompted us to analyse whether this derepression also leads to changes in the expression of the repeat elements. To this end, we analysed the RNA-seq data for repeat expression and identified differentially expressed repeats in all conditions. Resistance development independent of FLYWCH1 KD (Ctr+cPt versus Ctr) showed the smallest number of differentially expressed repeats (Supplementary Fig. S16A and Supplementary Table S11), while FLYWCH1 KD alone (KD versus Ctr) induced larger effects and led to more differentially expressed repeats (Supplementary Fig. S16B and Supplementary Table S12). Again, we observed the most profound effects for FLYWCH1 KD in conjunction with early resistance development (KD+cPt versus Ctr) (Fig. 7G and Supplementary Table S13). This implies that FLYWCH1 influences resistance development by regulating repeat expression. The largest effects on repeat expression were observed in long-term resistant cells (Supplementary Fig. S16C and Supplementary Table S14). We noticed that the extent of up- or downregulation in the expression of the repeats was comparable to that of deregulated genes, with similar ranges of fold changes (Supplementary Fig. S16D–F). In line with the loss of H3K9me3 at Alu elements (Fig. 7E), we discovered a gain in expression of a total of 23 Alu elements, with the strongest effect caused by FLYWCH1 KD alone (KD versus Ctr), followed by KD and early resistance development (KD+cPt versus Ctr) and resistance development (Ctr+cPt versus Ctr) on its own (Fig. 7H). As expected from its association with both regions that gain and lose H3K9me3 in resistant cells, Alu expression was much more variable in long-term resistant A2780cis cells, with a predominant downregulation of Alu expression (Fig. 7H). In line with the observed loss of H3K9me3 induced by FLYWCH1 KD and resistance development (Fig. 7E), the LTR class and especially the ERV1 family showed many elements with upregulated expression and even a set of elements that gain expression in FLYWCH1 KD as well as in both early and long-term resistance development (Fig. 7H).

In summary, FLYWCH1 suppression and early resistance development lead to alterations of H3K9me3 levels at repeat elements that are also deregulated in long-term resistance. This induces changes in the expression of SINE and LTR elements and thereby increases transcriptional plasticity.

## Discussion

The development of resistance to platinum-based chemotherapy still represents a significant challenge in the clinical management of patients with advanced EOC. Thus, there is an urgent unmet need to understand the process of resistance development in detail and to identify factors that regulate resistance acquisition. This consequently will allow identification of novel biomarkers and potential therapeutic targets that will ultimately aid to improve the prognosis and effective treatment of EOC patients.

Our study demonstrates that FLYWCH1 is an important regulator of platinum-resistance in EOC. EOC cells with low FLYWCH1 levels display improved survival to cPt treatment and loss of FLYWCH1 is connected to resistance development in EOC cells. Our data were generated in the EOC cell line A2780, which was established from a patient with ovarian endometroid adenocarcinoma. Although this cell line is a well-established and frequently used model for EOC research, it is important to note that most patient samples are high-grade serous carcinoma. Therefore, it is crucial to validate the findings in the clinical context and indeed, loss of FLYWCH1 was also associated with a decreased OS of patients with EOC (Fig. 1). This is in line with earlier findings that loss of FLYWCH1 induces tumour progression of CRC and AML [19, 20], suggesting that it is indeed an important player in tumour progression. We validated these findings in EOC PDOs, which are valuable preclinical models that recapitulate the heterogeneity and 3D spatial organization found in the original tumour and thus are important tools to complement and validate findings from cell lines [106]. These experiments could confirm that patients with low FLYWCH1 expression experienced early recurrence compared to patients with high expression of FLYWCH1. Interestingly, sensitive cells exposed to cPt for a prolonged time develop a resistant phenotype, which is accompanied by a reduction in FLYWCH1 expression even without the active reduction of FLYWCH1 by shRNA expression (Fig. 1). Whether this acquisition of resistance to cPt is caused by an active loss in FLYWCH1 expression through an unknown mechanism or if this is a selection process where cells with inherently low FLYWCH1 levels survive the treatment process remains to be clarified.

A recent study showed that FLYWCH1 is localized to but cannot directly interact with H3K9me3 [22]. Our data demonstrate that while FLYWCH1 and H3K9me3 occupy the same CSCs in sensitive A2780 cells and PDO lines, this colocalization is lost upon resistance development (Fig. 2). Interestingly, FLYWCH1 does not only colocalize with H3K9me3 in EOC cells but also with H3K27me2/me3 (Supplementary Fig. S3F). This implies that FLYWCH1 is not just linked to constitutive heterochromatin but might rather be a part of the silencing heterochromatic CSC in general.

Using proximity ligation, FLYWCH1 was shown to associate with H3K9me3 as well as various heterochromatic proteins such as CBX3 [22] and chromatin compaction by CBX3 is dependent on biomolecular condensation processes [107]. Besides two terminal IDRs, FLYWCH1 also possesses four potential internal IDRs and five zinc finger domains, which led to the hypothesis that FLYWCH1 associates with H3K9me3 loci through biomolecular condensation. While the C-terminal IDR of FLYWCH1 has the ability to form biomolecular condensates *in vitro*, neither of the terminal IDRs of FLYWCH1 was sufficient to induce the formation of nuclear condensates in living cells (Supplementary Fig. S5). Most likely, the zinc finger domains contribute to the foci formation of FLYWCH1, as multivalency plays an important role in the formation of biomolecular condensates [108]. Furthermore, a direct interaction of FLYWCH1 and CBX3 cannot be excluded and should be explored in follow-up studies. While resistance development led to a loss in FLYWCH1 and its colocalization with H3K9me3, acute treatment with cPt increased the levels of FLYWCH1 and H3K9me3 in the nucleus and led to an increase in colocalization. These changes might be explained by the concentration dependence of formation of biomolecular condensates, where condensates only form once the protein concentration exceeds a specific saturation concentration [109].

While FLYWCH1 and H3K9me3 levels are correlated in sensitive cells and loss of FLYWCH1 by shRNA KD also reduced H3K9me3 levels, we did not observe this trend in long-term resistant A2780cis cells, which lose FLYWCH1 and gain H3K9me3. We attribute this difference to the importance of FLYWCH1 during acute cPt treatment and resistance development but not playing an active role in resistance maintenance. This is supported by the observation that in A2780cis cells, FLYWCH1 overexpression did not show any effects on proliferation and no silencing in the reporter assay was observed (Fig. 5C, and Supplementary Fig. S1G and H). RNA-seq analysis demonstrated that recently identified putative coregulators of FLYWCH1 [22] are deregulated in the long-term resistant cell line A2780cis. Future work needs to focus on the clear determination of essential cofactors of FLYWCH1 in sensitive and resistant EOC cells.

Deposition of H3K9me3 is also observed at sites of acute DNA damage [89] and previously it was suggested that FLYWCH1 might be involved in the DNA damage response because it colocalizes with γH2A.X in untreated cells and the levels of both are increased upon UV treatment [21]. While we confirmed the colocalization of FLYWCH1 and γH2A.X in untreated cells, the new γH2A.X foci formed upon acute treatment with cPt displayed no colocalization with FLYWCH1. This implies that FLYWCH1 might have a role in the repair of endogenous DNA damage, rather than the resolution of double strand breaks. However, the role of FLYWCH1 in DNA damage repair seems to be a complex matter and dependent on many factors, including the DNA damage inducing agent and the p53 status of the cells [21]. Future studies investigating the potential involvement of FLYWCH1 with DNA damage repair and the specific pathways in PDOs will determine if FLYWCH1 has a direct role in DNA damage repair in patient-derived cells.

The observed alterations in colocalization of FLYWCH1 with H3K9me3 and the loss of FLYWCH1 expression in long-term resistant cells suggest a role for FLYWCH1 in regulating H3K9me3/H3K27me3 levels. Our study demonstrates that cells with low levels of FLYWCH1, either by enforced suppression or early or long-term resistance development, also display changes in H3K9me3 at distinct genomic loci (Figs 5F–I and Fig. 6). H3K9me3 is part of heterochromatic CSCs, where it is mainly involved in silencing repeat elements but is also found at genes [24], where it has different functions. Within the gene body, H3K9me3 can ensure proper transcription of genes and silence repeat elements, whereas when found at the transcription start site of genes, H3K9me3 can induce gene silencing [110]. We found that some of the regions with differential H3K9me3 signal in FLYWCH1 KD or resistance development coincided with promoters (Supplementary Fig. S14C and D). However, most of the identified differential regions were rather associated with repeat elements. Particularly, FLYWCH1 loss and resistance development led to a loss of H3K9me3 in LTR and Alu elements. These are associated with transcriptional plasticity, as both LTR and Alu elements can boost gene expression by acting as enhancers [33, 35] and SINE elements near promoters can induce transcriptional silencing [111]. The observed loss of H3K9me3 at Alu and LTR elements also led to reactivation of their expression in FLYWCH1 KD and early resistance development (KD, Ctr+cPt and KD+cPt) and in parts also in long-term resistant A2780cis cells. This once more highlights the dynamic nature of the transcriptional changes occurring during resistance development and is in line with our previous data that while some changes occur early in resistance development, others only establish during long-term resistance [16].

Analyses of the direct effect of FLYWCH1 on gene expression showed that recruitment of FLYWCH1 to a synthetic promotor induced gene silencing in sensitive A2780 but not resistant A2780cis cells (Fig. 5A–C). In line with this finding, overexpression of FLYWCH1 in resistant cells to mimic the expression levels of sensitive cells had no discernible effect, neither on platinum sensitivity (Supplementary Fig. S1F and G) nor on gene expression (Supplementary Fig. S10). This implies that loss of FLYWCH1 is important for the establishment of resistance rather than for its maintenance and that resistant cells downregulate not only FLYWCH1 but also important coregulators of FLYWCH1. In order to identify putative FLYWCH1 coregulators with altered expression in resistant A2780cis cells, we employed previously published data that identified FLYWCH1 proximal proteins [22]. These include many proteins that interact with H3K9me3, such as methyltransferases, zinc finger proteins and Polycomb proteins, but also transcriptional regulators such as JunB. We noticed that 16 out of 26 FLYWCH1 interaction partners were downregulated in expression in resistant cells (Supplementary Fig. S11G). While most of the putative interactors only showed mild effects in deregulation, JunB showed significant downregulation in A2780cis cells (Supplementary Fig. S11F and G). The proposed interaction of FLYWCH1 and JunB raises the question as to whether FLYWCH1 does not only induce gene silencing but might also be a bivalent factor that has silencing effects via its association with H3K9me3 and H3K27me3 but also activating effects via its interaction with JunB.

The alterations in H3K9me3 induced by the loss of FLYWCH1, either by enforced suppression or by development of platinum resistance, were accompanied by increased transcriptional plasticity and led to deregulation of many of the same genes but to different extents (Fig. 4). This suggests that loss of FLYWCH1 accelerates the resistance development process by deregulating pathways that are also affected by resistance development. DEGs in long-term resistant cells showed similar effects to DEGs in early resistance development and vice versa, but again to a lesser extent (Fig. 4G and H, and Supplementary Fig. S11B and C). This illustrates that resistance development is a highly dynamic process induced through transcriptional plasticity, which is in line with previous studies [12, 16, 112, 113]. While some genes are only deregulated during resistance development but bounce back once resistance is fully developed, others gradually change and stay deregulated in long-term resistant cells. Loss of FLYWCH1 and early resistance development, independent and dependent on FLYWCH1, induced the upregulation of Myc target genes and genes linked to oxidative phosphorylation. This is in line with previous studies that found that c-Myc is negatively correlated with FLYWCH1 expression [20] and that c-Myc is an important factor in ovarian cancers as it is upregulated in platinum resistant cells and associated with decreased disease-free and OS [114]. Oxidative phosphorylation is involved in the resistance development to different drugs and in different cancer types [91]. While platinum-sensitive ovarian cancer cells rely on glycolysis for energy production, resistant cells can switch between glycolysis and oxidative phosphorylation [115], providing them with a survival advantage and promoting metastasis formation [116]. Indeed, treatment of ovarian cancer cells with oxidative phosphorylation inhibitors can resensitize platinum-resistant cells [117], and there are indications that it might prevent resistance development [118]. Increased oxidative phosphorylation caused by loss of FLYWCH1 might therefore be an important factor in promoting resistance development. While FLYWCH1 suppression and early resistance development induced upregulation of pathways linked to resistance development, they also led to the downregulation of apoptosis, immune response, inflammation, and hypoxia pathways, all of which have been shown to be downregulated in early platinum resistance of EOC cells before [119]. Deregulation of apoptosis is a known phenomenon during development of platinum resistance [120–122] and helps the cell evade drug treatment. While acute inflammation can induce an anti-tumour immune response, chronic inflammation can be induced by therapeutic treatment and is typically known to promote tumour progression and resistance [123, 124]. In line with this, long-term resistance development led to an upregulation of the interferon response.

Although FLYWCH1 was recently suggested to be a negative regulator of Wnt signalling in AML and CRC [19, 20], we did not detect upregulation in Wnt signalling upon loss of FLYWCH1 or early resistance development in EOC (Supplementary Fig. S2E and F) but rather downregulation of Wnt signalling genes (Supplementary Fig. S9). Recent data showed that treatment of platinum-resistant A2780cis cells with inhibitors of Wnt signalling improves platinum-sensitivity [43, 125]. Thus, Wnt signalling might be a pathway that becomes activated later during the resistance development process or in maintenance of the resistant state.

In conclusion, our study demonstrates that FLYWCH1 is a critical regulator of platinum-resistance in EOC and proposes a model, in which FLYWCH1 is associated with H3K9me3 in sensitive A2780 cells, where it is involved in silencing of genes and repeat elements. Loss of FLYWCH1 leads to loss of H3K9me3 at LTR and SINE elements and deregulation of H3K9me3 at promotor regions, which influences the expression of genes and repeats. We hypothesize that the derepression of repeat elements caused by FLYWCH1 suppression leads to several effects. First, while some derepressed repeats can activate gene expression by functioning as enhancers [33], others might act at transcriptional repressors, as shown for SINE elements [111]. Additionally, loss of H3K9me3 at repeat elements leads to the re-expression of previously silenced repeats, which can hijack the transcription machinery, leading to a reduction in the expression of certain genes. This effect has been previously observed in embryonic stem cells after depletion of the TRIM28 heterochromatin corepressor [36]. Loss of the transcriptional machinery from actively described genes leads to their downregulation and renders the promoter vulnerable to the binding of transcriptional repressors and silencing factors, which could explain the gain in H3K9me3 at promotor regions observed upon suppression of FLYWCH1. The combined action of repeat deregulation and the disrupted transcription of cellular identity genes increases cellular plasticity and leads to alteration of pathways that enhance the development of platinum resistance. This data-driven hypothesis explains the bivalent effect we see in cells upon FLYWCH1 KD and explains why loss of FLYWCH1 can accelerate resistance development in EOC. Furthermore, our findings propose FLYWCH1 as a potential biomarker for treatment response and prognosis of patients with EOC.

## Supplementary Material

zcaf012_Supplemental_Files

## Data Availability

NGS data in this publication are available via the NCBI’s Gene Expression Omnibus [126]. RNA-seq data are accessible via the GEO Series accession number GSE273904 (https://www.ncbi.nlm.nih.gov/geo/query/acc.cgi?&acc=GSE273904) and H3K9me3 ChIP-seq data via the GEO accession number GSE273903 (https://www.ncbi.nlm.nih.gov/geo/query/acc.cgi?acc=GSE273903).
