# Supplementary material for: Identification of FLYWCH1 as a regulator of platinum-resistance in epithelial ovarian cancer
Source: NAR Cancer. 2025 Apr 4;7(2):zcaf012. doi: 10.1093/narcan/zcaf012 (PMC11970373; doi:10.1093/narcan/zcaf012)
Supplement: zcaf012_Supplemental_Files [file zcaf012_supplemental_files.zip › Supplementary_material.pdf]

Identification of FLYWCH1 as a regulator of platinum-resistance in epithelial ovarian cancer

Tabea L. Fullstone[1], Helene Rohm[1], Till Kaltofen[2,3], Sophia Hierlmayer[2], Juliane Reichenbach[2], Simon Schweikert[1], Franziska Knodel[1], Ann-Kathrin Loeffler[1], Doris Mayr[4], Udo Jeschke[2,5], Sven Mahner[2], Mirjana Kessler[2], Fabian Trillsch[2] and Philipp Rathert[1,*]

[1] Department of Biochemistry, Institute of Biochemistry and Technical Biochemistry, University of Stuttgart, 70569 Stuttgart, Germany

[2] Department of Obstetrics and Gynaecology, University Hospital, LMU Munich, 81377 Munich, Germany;

[3] Department of Surgery, University Hospital Regensburg, 93053 Regensburg, Germany;

[4] Institute of Pathology, LMU Munich, 81377 Munich, Germany;

[5] Department of Obstetrics and Gynaecology, University Hospital Augsburg, 86156 Augsburg, Germany

* To whom correspondence should be addressed. Tel: +49 711 685 64388; Fax: +49 711 685 64392; Email: philipp.rathert@ibtb.uni-stuttgart.de

Supplementary Material

Supplementary Figures 4

Figure S1. Loss of FLYWCH improves proliferation of SKOV3 but not resistant A2780cis cells. . 4

Figure S2. FLYWCH1 is independent of Wnt signalling and FLYWCH1 levels are reduced in resistant OC cell lines and in patients with high cancer stages. 5

Figure S3. FLYWCH1 is associated with H3K9me and H3K27me2/me3. 6

Figure S4. Colocalisation of FLYWCH1 and H3K9me3 is reduced in PDO from patients that experience with recurrence or death........ 7

Figure S5. Hexanediol dissolves FLYWCH1 foci in the nucleus and leads to loss of colocalization with H3K9me3. 8

Figure S6. Cisplatin treatment leads to increased FLYWCH1 expression and H3K9me3 levels, 10

Figure S7. FLYWCH1 is not associated with acute DNA damage response. 12

Figure S8. FLYWCH1 KD enhances the differential regulation of genes associated with resistance development........ 14

Figure S9. Loss of FLYWCH1 and early resistance development lead to the deregulation of pathways associated with resistance development........ 15

Figure S10. Overexpression of FLYWCH1 in resistant A2780cis cells has no significant effect on gene expression. 16

Figure S11. Resistance development is associated with changes in gene expression of epigenetic factors including putative FLYWCH1 interacting proteins. 17

Figure S12. Resistant A2780cis cells show changes in H3K9m3 signal. 18

Figure S13. FLYWCH1 knockdown and early resistance development are associated with changes in H3K9me3. 19

Figure S14. FLYWCH1 knockdown and resistance development lead to changes in H3K9me3 at repeat elements........ 20

Figure S15. FLYWCH1 knockdown and resistance development are associated with changes at LTR and SINE elements........ 22

Figure S16. FLYWCH1 knockdown and resistance development leads to changes in repeat expression to a comparable level to the gene expression changes. 23

Supplementary Tables are available in data file SupplementaryTablesS1-S10.xlsx:

Supplementary Table S1: shRNAs used in this study.

Supplementary Table S2: Primers used for RT-qPCR

Supplementary Table S3: Clinical characteristics of the patients from which the organoids were derived.

Supplementary Table S4: Statistical significance for gene expression changes in FLYWCH1 knockdown vs control (KD vs Ctr) determined by DeSeq2.

Supplementary Table S5: Statistical significance for gene expression changes in early resistance development vs control (Ctr+cPt vs Ctr) determined by DeSeq2.

Supplementary Table S6: Statistical significance for gene expression changes in early resistance development with the aid of FLYWCH1 suppression vs control (KD+cPt vs Ctr) determined by DeSeq2.

Supplementary Table S7: Gene-set-enrichment analysis (GSEA) for Hallmark pathways on all significantly up- or downregulated genes for each condition.

Supplementary Table S8: Statistical significance for gene expression changes in A2780cis cells with reintroduction of FLYWCH1 expression vs expression of 3XFLAG as control determined by DeSeq2.

Supplementary Table S9: Statistical significance for gene expression changes in platinum resistant vs platinum sensitive cells (A2780cis vs A2780) determined by DeSeq2.

Supplementary Table S10: Gene-set-enrichment analysis (GSEA) for Hallmark pathways on all significantly up- or downregulated genes in resistant A2780cis vs sensitive A2780 cells.

Supplementary Table S11: Statistical significance for repeat expression changes in early resistance development vs control (Ctr+cPt vs Ctr) determined by DeSeq2.

Supplementary Table S12: Statistical significance for repeat expression changes in FLYWCH1 knockdown vs control (KD vs Ctr) determined by DeSeq2.

Supplementary Table S13: Statistical significance for repeat expression changes in early resistance development with the aid of FLYWCH1 suppression vs control (KD+cPt vs Ctr) determined by DeSeq2.

Supplementary Table S14: Statistical significance for repeat expression changes in platinum resistant vs platinum sensitive cells (A2780cis vs A2780) determined by DeSeq2.

Supplementary Figures

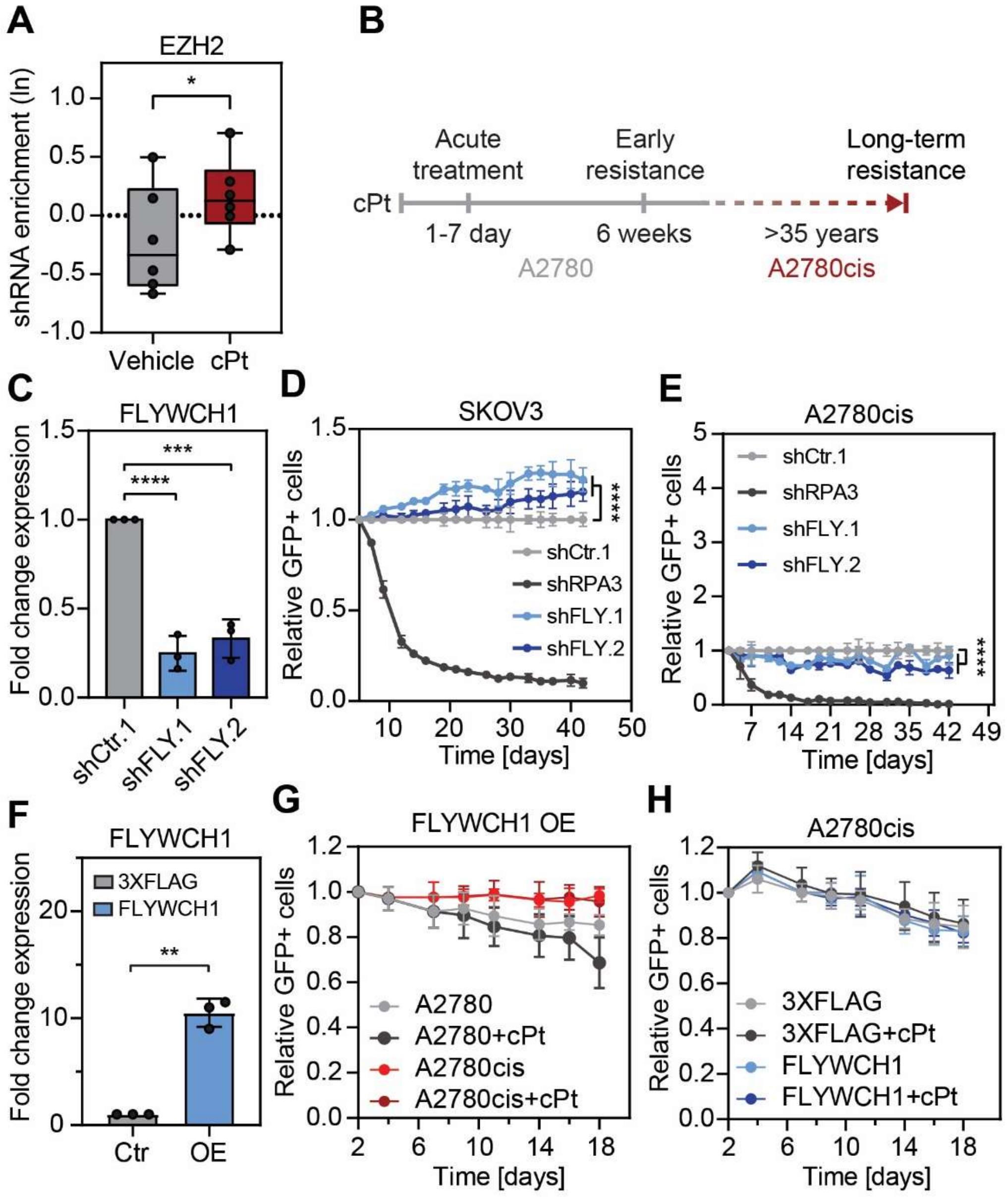

Figure S1. Loss of FLYWCH improves proliferation of SKOV3 but not resistant A2780cis cells.
(A) Enrichment of cells expression one of six different shRNAs targeting EZH2 and treated with either 1µM cPt or vehicle. shRNA enrichment was calculated relative to shRNAs in the original pools. Data is shown as a box plot with all data points displayed. Statistical analysis: paired t-test (*: p≤0.05). **(B)** Timeline of the cPt treatment scheme employed in this study. Acute treatment corresponds to 1-7 days of treatment with 1 µM cPt. Early resistance develops after treatment of sensitive A2780 cells with 1 µM cPt for 6 weeks. A2780cis cells have been established >35 year ago and constitute long-term resistant cells. **(C)** Gene expression of FLYWCH1 (RT-qPCR) relative to SDHA control and shCtr.1. FLYWCH1 knockdown was induced in SKOV3 cells for 10 weeks with two independent shRNAs targeting FLYWCH1. Mean±SD, n=3, statistical analysis: ordinary one-way ANOVA and Dunnett's multiple comparisons test (***: p≤0.001, ****: p≤0.0001). **(D and E)** Competitive proliferation assays of SKOV3 (D) and A2780cis (E) cells expressing the indicated shRNAs to determine the effects of suppression of FLYWCH1 expression on cell proliferation. Shown is the fraction of shRNA+/GFP+ cells relative to the initial measurement and shCtr.1. Mean±SD, n=3, statistical analysis: two-way ANOVA and Dunnett's multiple comparisons test (****: p≤0.0001). **(F)** Gene expression of FLYWCH1 (RT-qPCR) relative to SDHA control and 3XFLAG. Expression of FLYWCH1 or 3XFLAG was induced in A2780cis cells for 7 days. Mean±SD, n=3, statistical analysis: unpaired t-test with Welch's correction (**: p≤0.01). **(G and H)** Competitive proliferation assays of A2780 and A2780cis cells expressing 3XFLAG or FLYWCH1 to determine the effects of FLYWCH1 overexpression on cell proliferation. Cells were cultivated in the presence of vehicle or 1 µM cPt from day 7 until the end of the assay. G shows the fraction of

shRNA+/GFP+ cells relative to the initial measurement and 3XFLAG. H shows the fraction of shRNA+/GFP+ cells relative to the initial measurement only. Mean±SD, n=3.

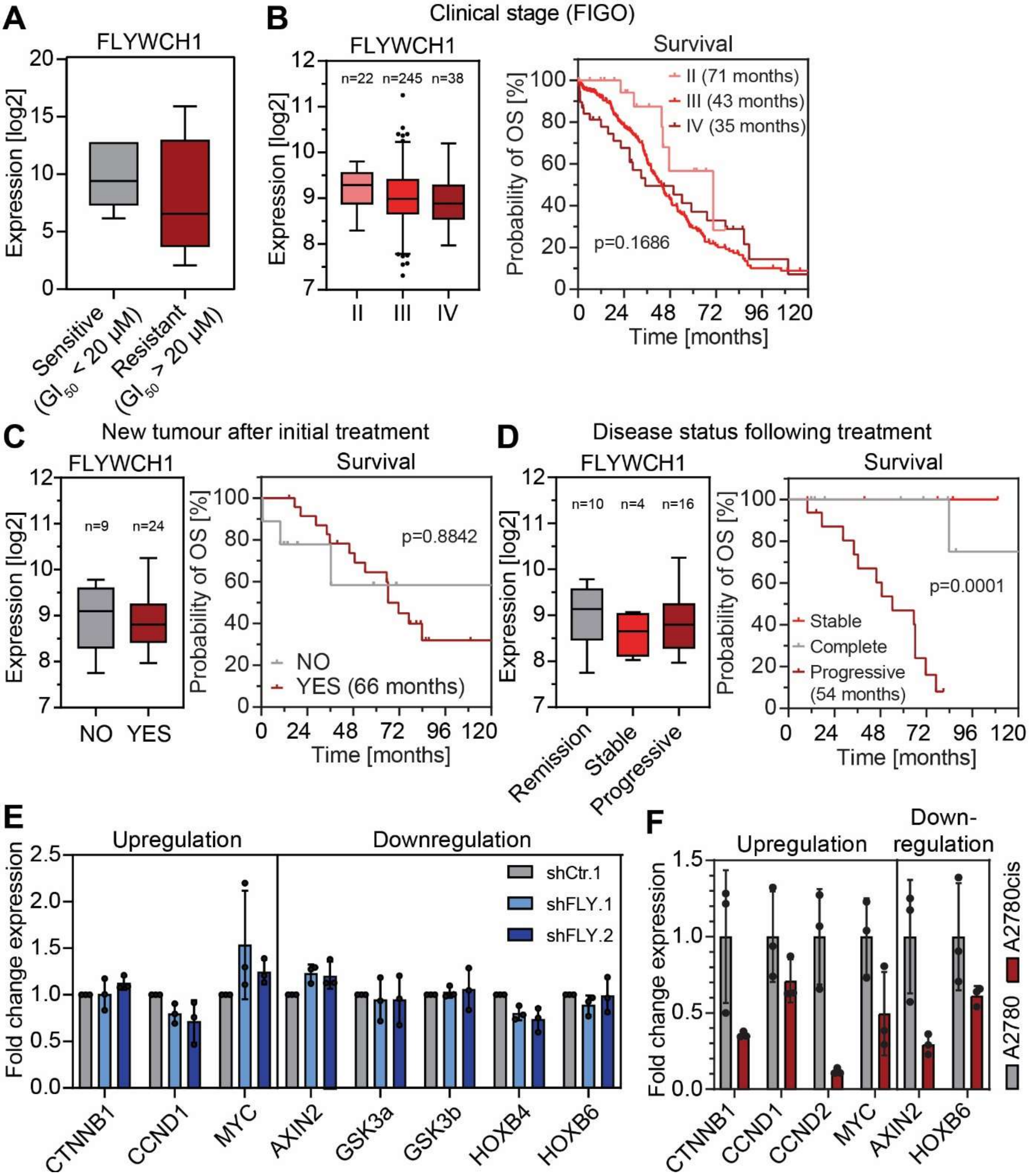

Figure S2. FLYWCH1 is independent of Wnt signalling and FLYWCH1 levels are reduced in resistant OC cell lines and in patients with high cancer stages.

(A) FLYWCH1 expression (RPKM) of cPt-sensitive and -resistant EOC cells lines curated from expression data for EOC cell lines (CCLE). Cells were divided depending on their GI_{50} value into sensitive ($GI_{50} \leq 20$, n=7) and resistant ($GI_{50} > 20$, n=11) cells. Box plot with 2.5 and 97.5 percentiles. **(B-D)** Analysis of FLYWCH1 expression (RNA-seq) and overall survival (OS) in patients with OC from the TCGA Ovarian Cancer (OV) cohort. Patients were grouped based on their clinical stage (B), occurrence of a new tumour event after initial treatment (C) or the disease status following treatment (D). Box plot with 2.5 and 97.5 percentile and Kaplan-Meier curve are shown for the indicated number of patient samples per group. Median survival of each group is indicated were applicable. Statistical analysis: Chi-Square statistics of the Log-Rank test (Mantel-Cox). **(E and F)** Gene expression (RT-qPCR) of Wnt signalling associated genes in SKOV3 cells following FLYWCH1 knockdown for 10 weeks (E) or A2780 and A2780cis cells (F). Data is shown relative to SDHA control and shCtr (E) or A2780 (F). Genes were categorized into expected up- or downregulated, based on the assumption that FLYWCH1 is a negative regulator of Wnt signalling. Mean±SD, n=3.

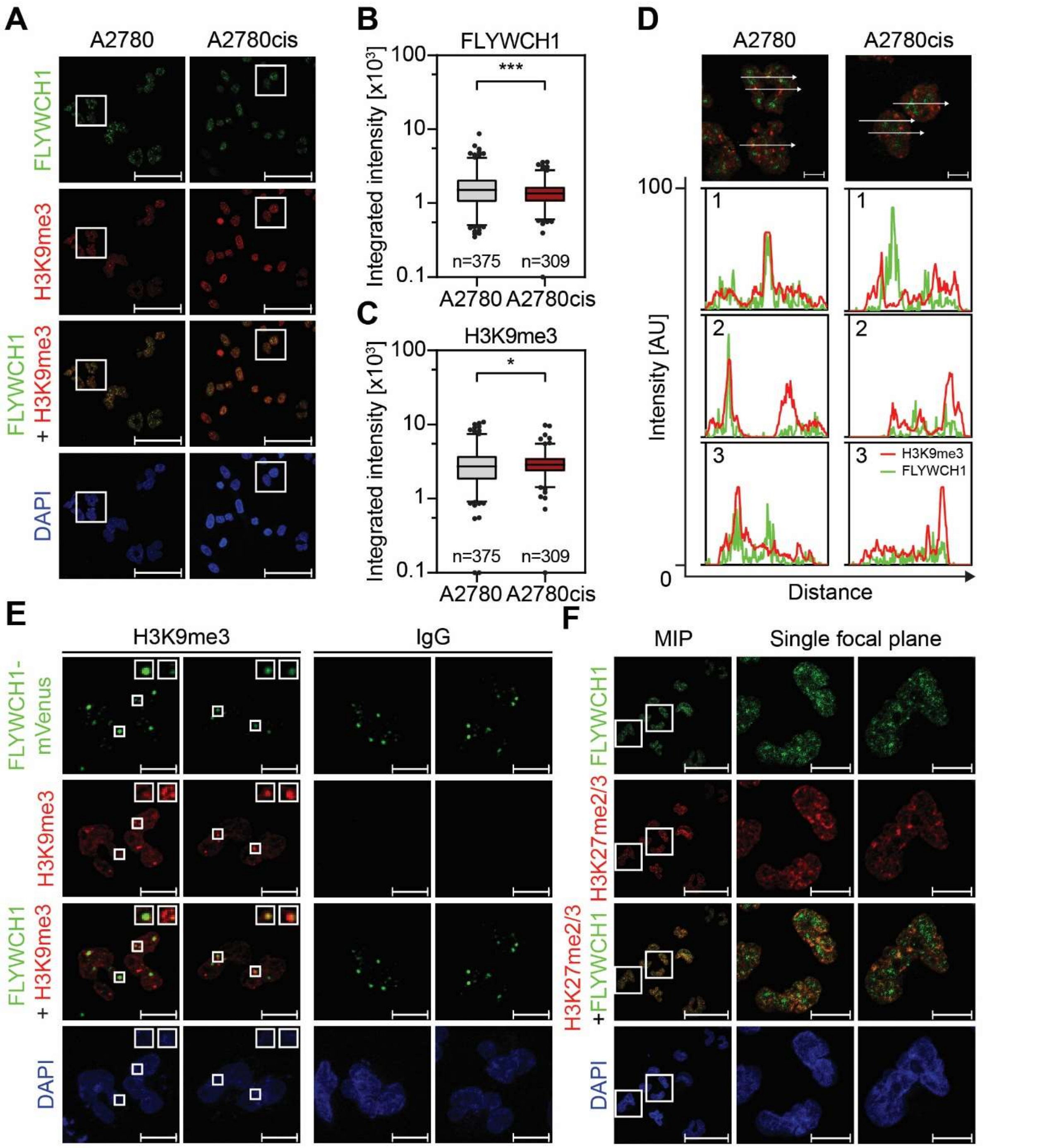

Figure S3. FLYWCH1 is associated with H3K9me and H3K27me2/me3.
(A) Representative maximum intensity projections of immunofluorescence staining of FLYWCH1 and H3K9me3. Sensitive A2780 and resistant A2780cis cells were fixed and then stained with the indicated antibodies. Boxes indicate cells show in the main figure (Fig. 2). Scale bar: 50 µm. **(B and C)** Quantification of maximum-intensity-projections of immunostaining images in A using CellProfiler. Integrated intensity of FLYWCH1 (B) or H3K9me3 (C) was analysed from the indicated number of nuclei. Box plot with 2.5 and 97.5 percentiles, n=3, statistical analysis: Mann-Whitney U test (*: p≤0.05, ***: p≤0.001). **(D)** Profile analysis of FLYWCH1 and H3K9me3 of single focal plane immunostaining images from A2780 and A2780cis cells. Intensity profiles along the indicated arrows were extracted using ImageJ. **(E)** Representative maximum intensity projections of ectopically expressed FLYWCH1 fused to mVenus and immunostaining for H3K9me3 or IgG control. Insets show enlarged images of the indicated foci. Scale bar: 10 µm. **(F)** Representative maximum intensity projection (MIP) and single focal plane images of immunofluorescence staining of FLYWCH1 and H3K27me2/me3. A2780 cells were fixed and then stained with the indicated antibodies. Boxes show cells depicted in the single focal plane. Scale bar MIP: 50 µm, scale bar single focal plane: 10 µm, n=3.

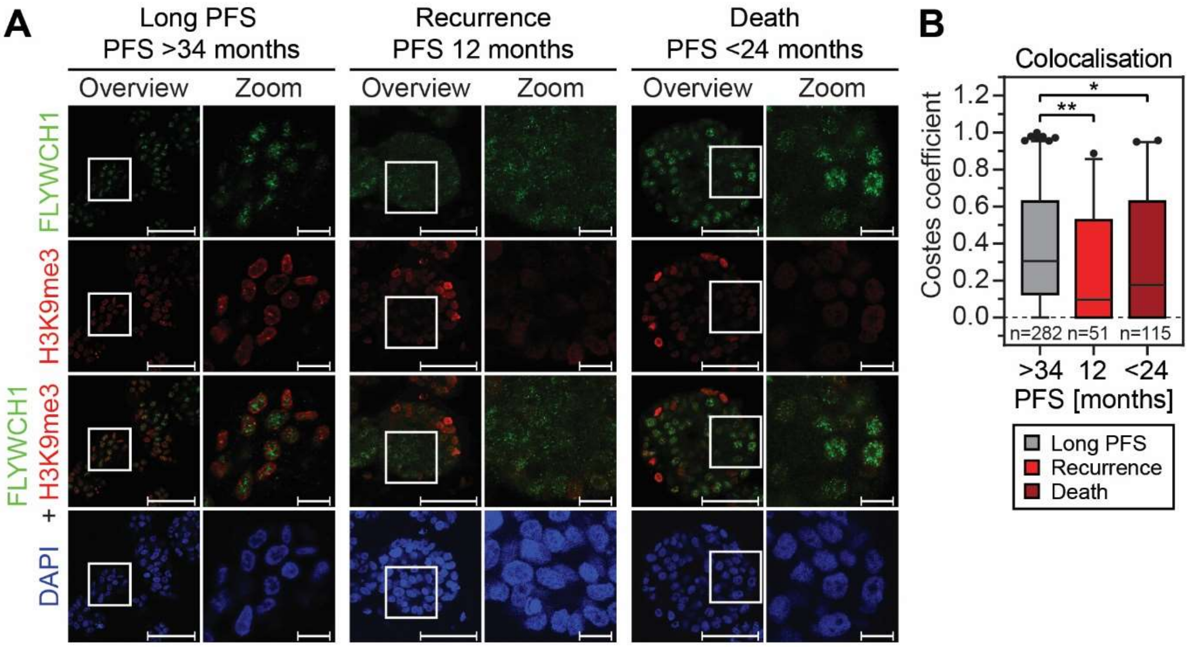

Figure S4. Colocalisation of FLYWCH1 and H3K9me3 is reduced in PDO from patients that experience with recurrence or death.
(A) Representative single focal plane images of immunofluorescence staining of FLYWCH1 and H3K9me3 in in three EOC PDOs derived from different patients with variable PFS times and treatment outcomes. PDOs were fixed and then stained with the indicated antibodies. Scale bar overview: 50 µm, scale bar zoom: 10 µm. **(B)** Quantification of colocalisation of FLYWCH1 and H3K9me3 from single focal plane immunofluorescence images in A using the Costes correlation coefficient. Analysis was performed on the indicated number of nuclei. Box plot with 2.5 and 97.5 percentiles, statistical analysis: Kruskal-Wallis test and Dunn's multiple comparisons test (*: $p \leq 0.05$, **: $p \leq 0.01$).

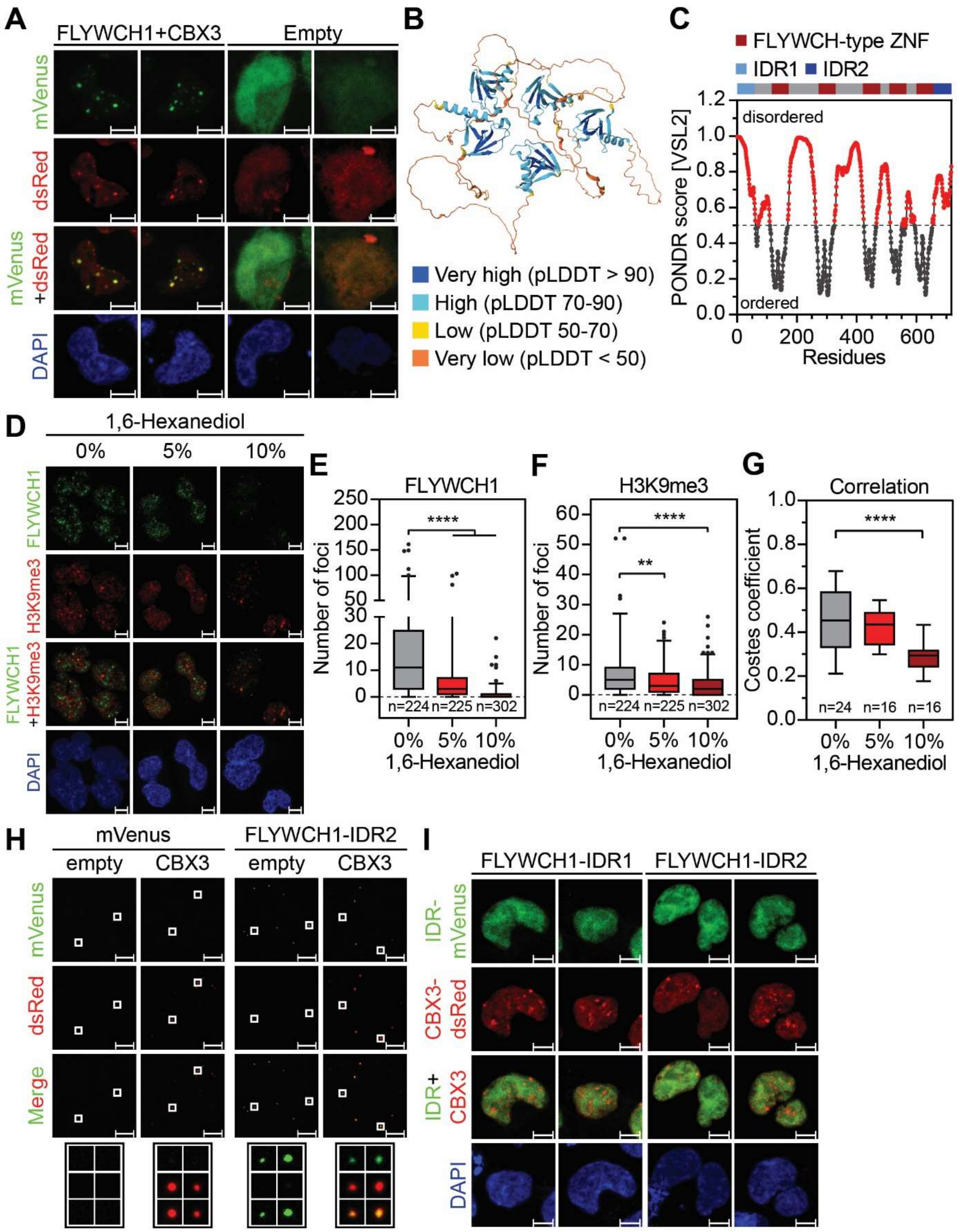

Figure S5. Hexanediol dissolves FLYWCH1 foci in the nucleus and leads to loss of colocalization with H3K9me3.
(A) Representative maximum intensity projections of ectopically expressed FLYWCH1-mVenus and CBX3-dsRed in A2780 cells. Empty dsRed and mVenus vectors were used as control. A2780 cells were transfected with the indicated constructs and after 24h cells were fixed and imaged. Scale bar: 10 µm, n=3. **(B)** Prediction of FLYWCH1 structure (AF-Q4VC44-F1-v4) from AlphaFold (https://alphafold.ebi.ac.uk/). Per-residue model confidence score (pLDDT) is indicated, where a pLDDT <50 suggests unstructured regions. **(C)** PONDR score of FLYWCH1 using the VSL2 algorithm (http://www.pondr.com/). Residues with a score > 0.5 are likely to be disordered and could be part of intrinsically disordered regions (IDRs). Residues with a score < 0.5 are likely to be part of structured domains. Above the graph, the domain structure of FLYWCH1 is shown based on UniProt annotations for FLYWCH1 (Q4VC44). The five structured FLYWCH-type zinc

finger domains are shown in red. Putative IDRs of FLYWCH1 used in downstream experiments are shown in blue. **(D)** Representative maximum intensity projections of immunofluorescence staining of FLYWCH1 and H3K9me3 following hexanediol treatment. A2780 cells were treated with 0%, 5% or 10% of 1,6-hexanediol for 10 mins, fixed and then stained with the indicated antibodies. Scale bar: 5 µm. **(E and F)** Quantification of FLYWCH1 (E) and H3K9me3 (F) foci from maximum-intensity-projections of immunostaining images in D using CellProfiler. Analysis was performed on the indicated number of nuclei. Box plot with 2.5 and 97.5 percentiles, n=2, statistical analysis: Kruskal-Wallis test and Dunn's multiple comparisons test (****: $p \leq 0.0001$). **(G)** Quantification of colocalisation of FLYWCH1 and H3K9me3 from single focal plane immunofluorescence images in D using the Costes correlation coefficient. Analysis was performed on the indicated number of images. Date shown is the average Costes coefficient per image, averaged over all z-stacks. Box plot with 2.5 and 97.5 percentiles, n=2, statistical analysis: Kruskal-Wallis test and Dunn's multiple comparisons test (****: $p \leq 0.0001$). **(H)** Representative images of in-vitro co-compartmentalisation experiments of FLYWCH1.IDR2 and CBX3. FLYWCH1-mVenus and CBX3-dsRed were incubated in combination or with fluorophore alone in crowding buffer for 20 min and then imaged to analyse droplet formation. Scale bar: 10 µm, n=2. **(I)** Representative maximum intensity projections of co-transfections of putative IDRs of FLYWCH1 and CBX3 in A2780 cells. A2780 cells were transfected with the indicated constructs and after 24h cells were fixed and imaged. Scale bar: 10 µm, n=2.

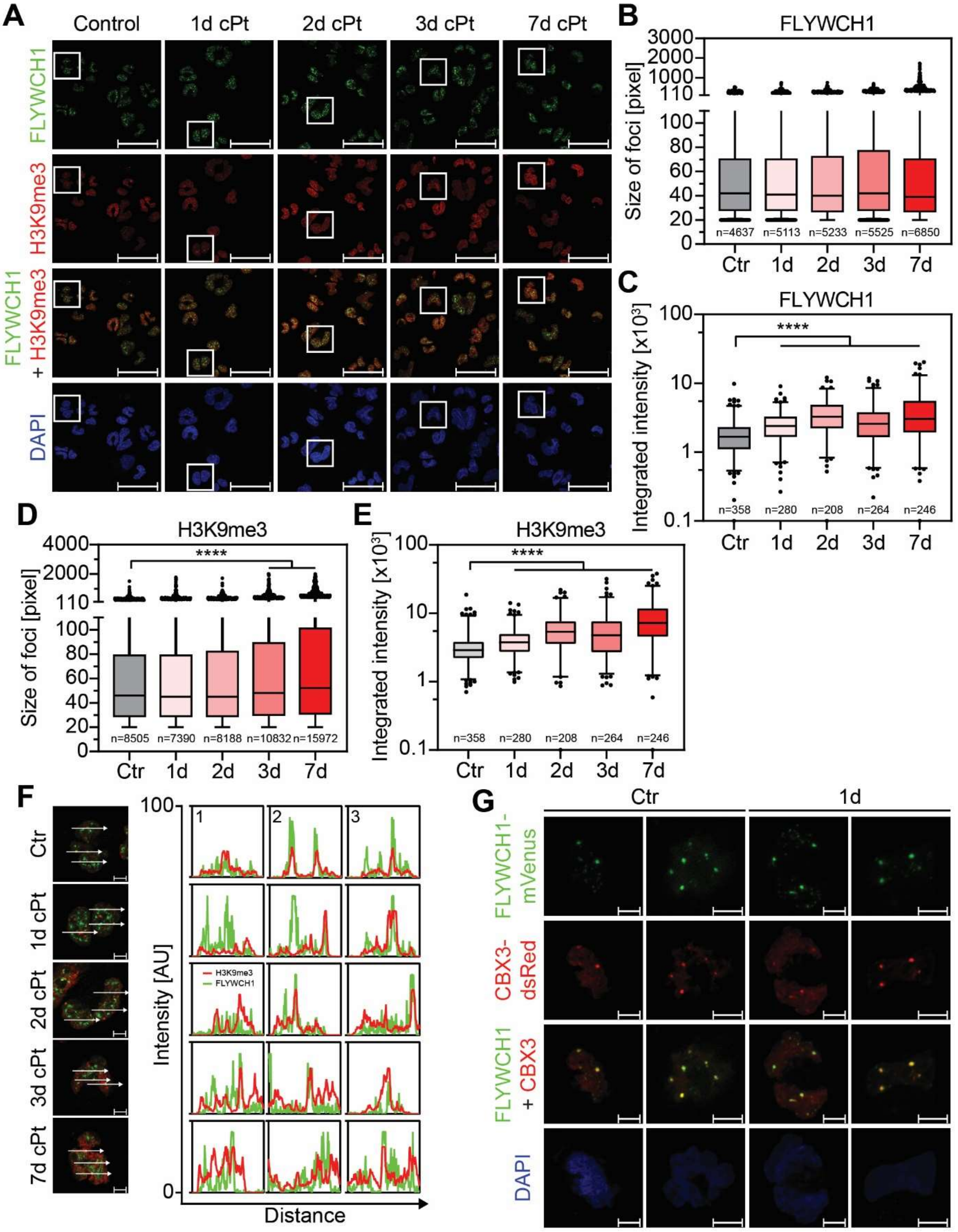

Figure S6. Cisplatin treatment leads to increased FLYWCH1 and H3K9me3 levels.
(A) Representative maximum intensity projections of immunofluorescence staining of FLYWCH1 and H3K9me3. Sensitive A2780 were treated with 1 µM cPt for the indicated time points. Cells were fixed and stained with the indicated antibodies. Boxes indicate cells shown in the main figure (Fig. 3A). Scale bar: 50 µm. **(B-E)** Quantification of maximum-intensity-projections of immunostaining images in A using CellProfiler. Analysis was performed on the indicated number of foci (B and D) or nuclei (C and E). Box plot with 2.5 and 97.5 percentiles, n=3, statistical analysis: Kruskal-Wallis test and Dunn's multiple comparisons test (****: $p \leq 0.0001$). (B and C) Quantification of spot area (B) and integrated intensity (C) of FLYWCH1 foci. (D and E) Quantification of spot area (D) and integrated intensity (E) of H3K9me3 foci. **(F)** Profile analysis of FLYWCH1 and H3K9me3 of single focal plane immunostaining images from A2780 cells treated with cPt for different time points. Intensity profiles along the indicated arrows were extracted using ImageJ. **(G)**

Representative maximum intensity projections of co-transfections of FLYWCH1-mVenus and CBX3-dsRed in A2780 cells treated with cPt or vehicle. A2780 cells were transfected with the indicated constructs and treated with 1 µM cPt or vehicle. After 24h cells were fixed and imaged. Scale bar: 5 µm, n=2.

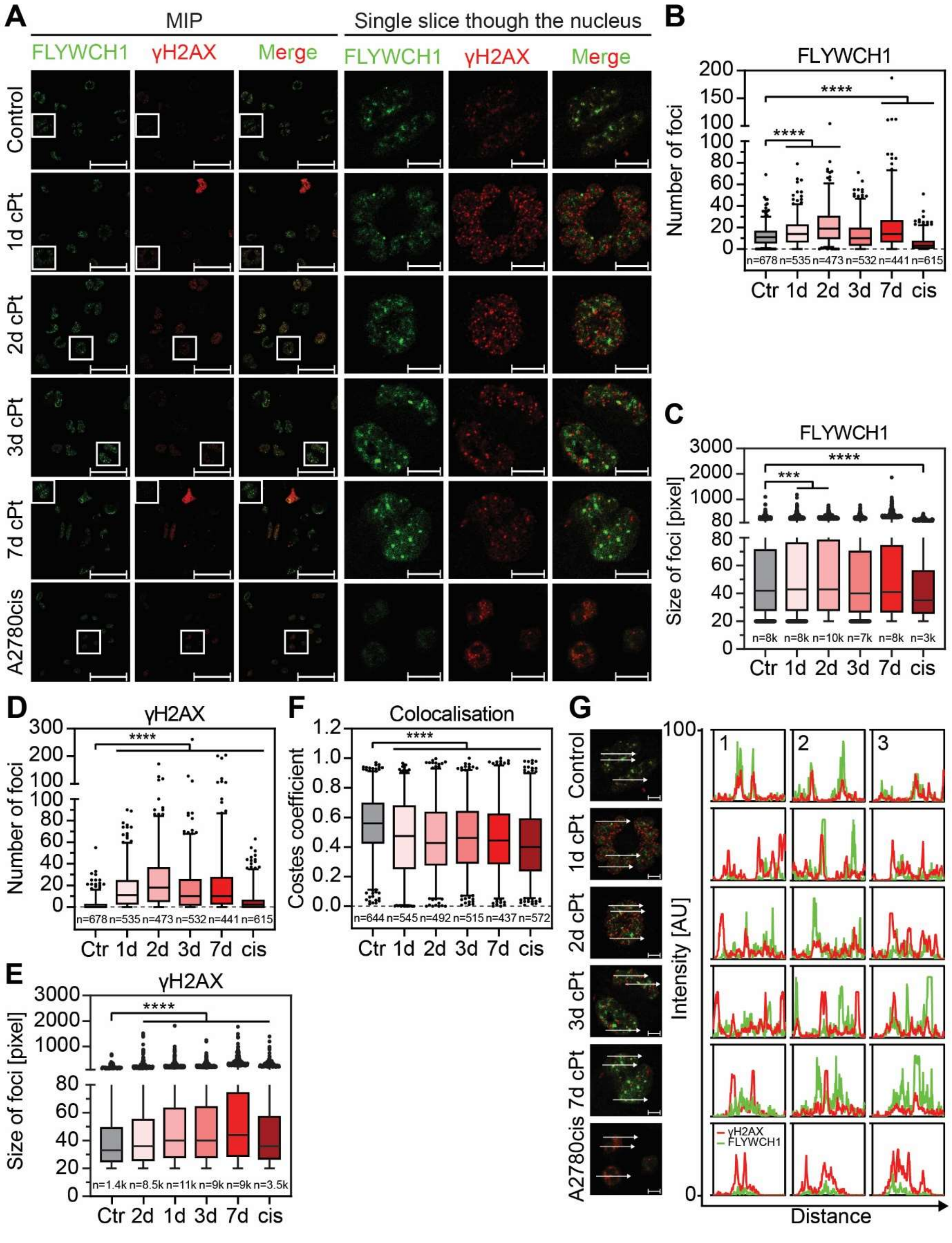

Figure S7. FLYWCH1 is not associated with acute DNA damage response.
(A) Representative maximum intensity projections (MIP) and single focal plane of immunofluorescence staining of FLYWCH1 and γH2A.X. Sensitive A2780 were treated with 1 µM cPt for the indicated time points. A2780 and A2780cis cells were then fixed and stained with the indicated antibodies. Boxes in the MIP panel indicate cells shown in the single plane images. Scale bar MIP: 50 µm, scale bar single slice: 10 µm. **(B-F)** Analysis of maximum-intensity-projections (B-E) or single focal plane immunofluorescence images (F) in A using CellProfiler. Analysis was performed on the indicated number of nuclei (B, D and F) or foci (C and E). (B and C) Quantification of number (B) and area (C) of FLYWCH1 foci. (D and E) Quantification of number (D) and area (E) of γH2A.X foci. (F) Quantification of colocalisation of FLYWCH1 and γH2A.X using the

Costes correlation coefficient. Box plot with 2.5 and 97.5 percentiles, n=3, statistical analysis: Kruskal-Wallis test and Dunn's multiple comparisons test (***: $p \leq 0.001$, ****: $p \leq 0.0001$). **(G)** Profile analysis of FLYWCH1 and γH2A.X from single focal plane immunostaining images of A2780cis cells and A2780 cells treated with cPt for different time points. Intensity profiles along the indicated arrows were extracted using ImageJ.

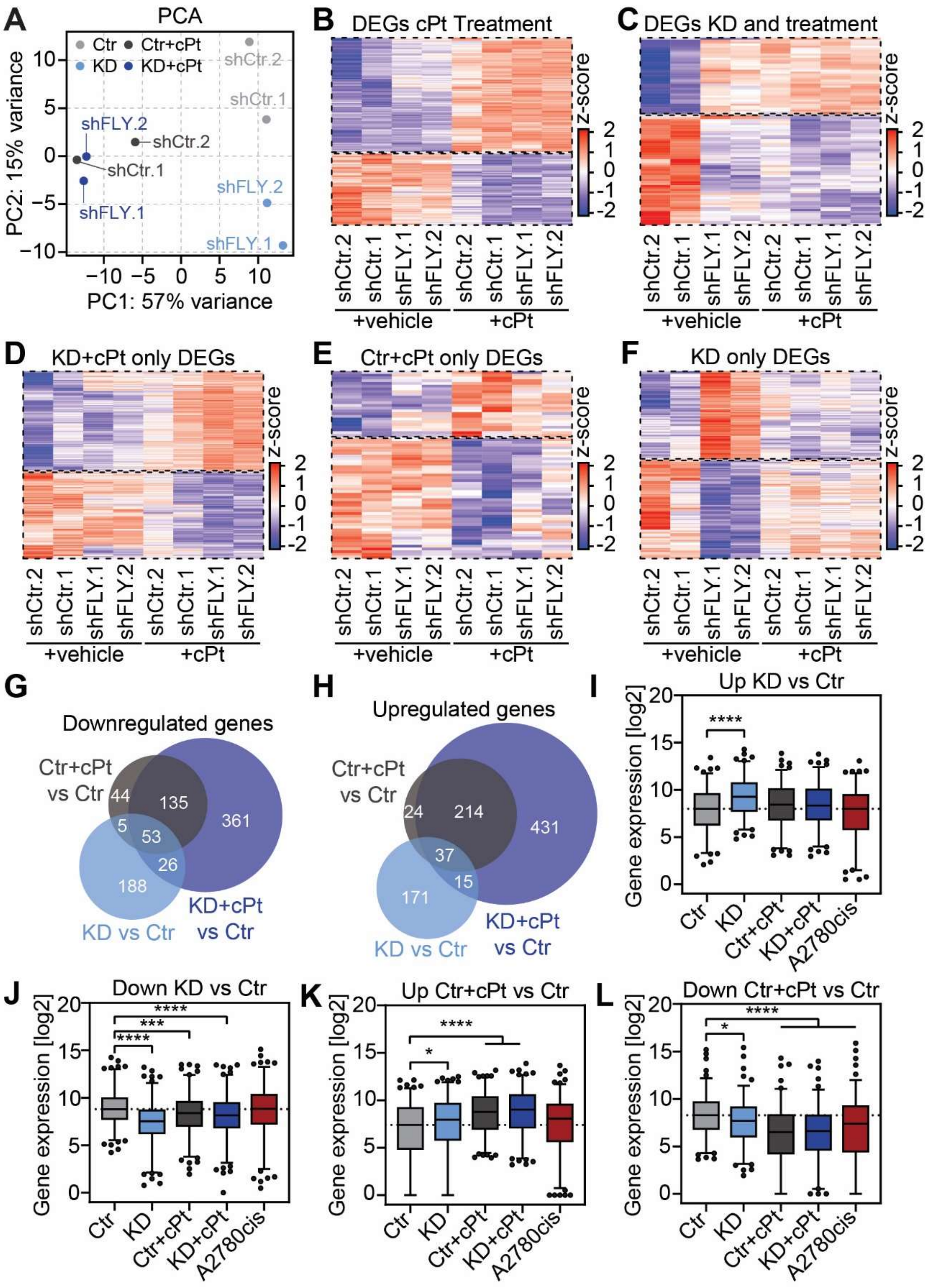

Figure S8. FLYWCH1 KD enhances the differential regulation of genes associated with resistance development.
(A) Principle component analysis of RNA-seq data on cells expressing two independent control and FLYWCH1 shRNAs with and without early resistance development by cPt treatment. **(B-F)** Zoom-in on clusters of differentially expressed genes (DEGs) from Fig. 4D. Groups were split into up- and down-regulated groups by k-means clustering. **(G and H)** Venn diagrams showing the overlap of differentially up- or downregulated genes in FLYWCH1 knockdown (KD vs Ctr), early resistance development (Ctr+cPt vs Ctr) and early resistance development with the aid of FLYWCH1 loss (KD+cPt vs Ctr). **(I-L)** Average expression of DEG in KD vs Ctr (**I** and **J**) and Ctr+cPt vs Ctr (**K** and **L**). Normalised count was averaged over the two independent shRNAs/repeats and plotted for each condition. Box plot with 2.5 and 97.5 percentiles,

statistical analysis: Kruskal-Wallis test and Dunn's multiple comparisons test (*: p≤0.05, ***: p≤0.001, ****: p≤0.0001).

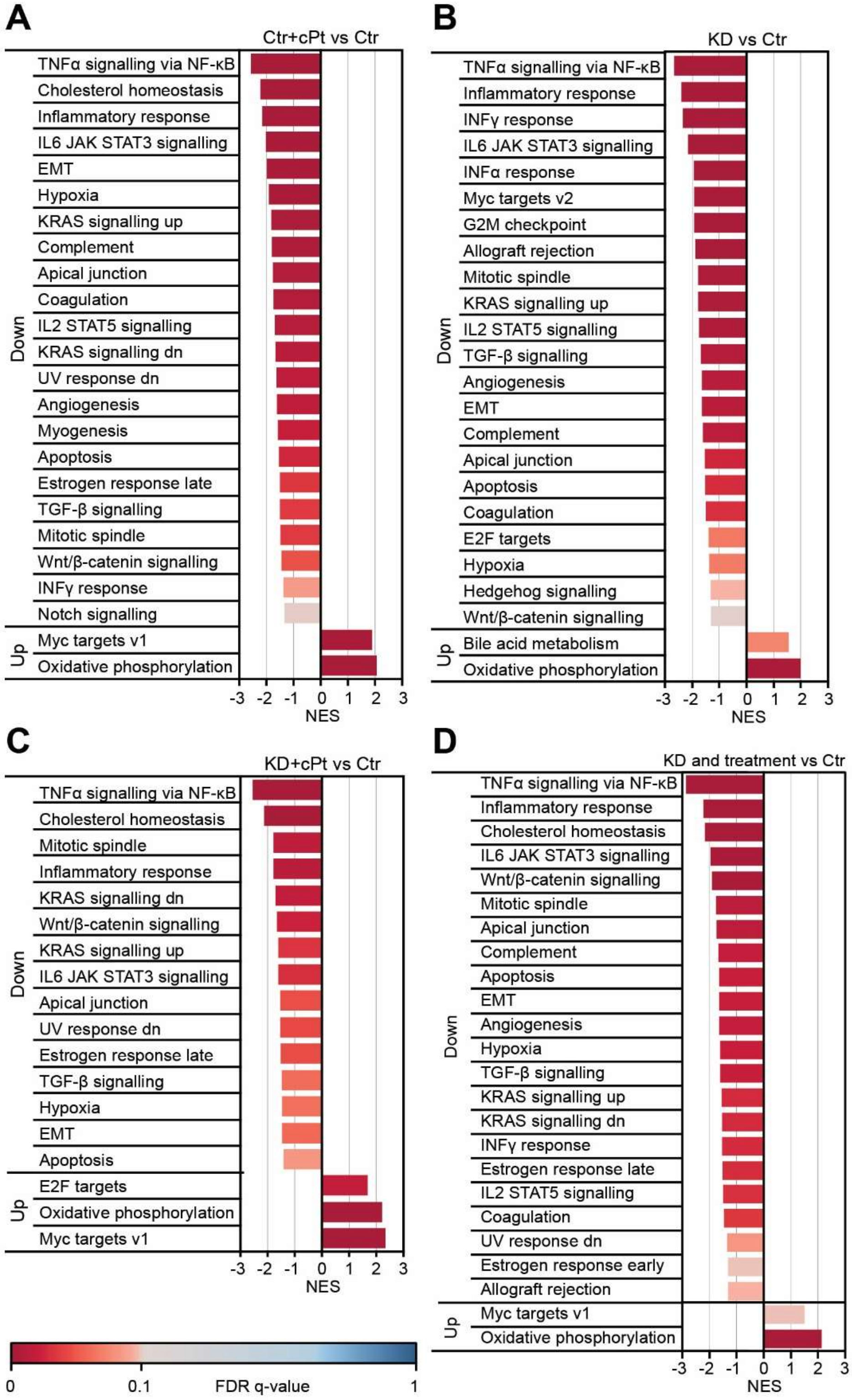

Figure S9. Loss of FLYWCH1 and early resistance development lead to the deregulation of pathways associated with resistance development.

(A-D) Gene-set-enrichment analysis for Hallmark pathways on all significantly up- or downregulated genes for each condition. In each panel, all significantly enriched pathways (FDR ≤ 0.1) are shown. Panels show pathways associated with genes deregulated in early resistance development (A), by FLYWCH1 suppression (B) by early resistance development with the aid of FLYWCH1 loss (C) and genes that are deregulated both upon suppression of FLYWCH1 and in early resistance development (D).

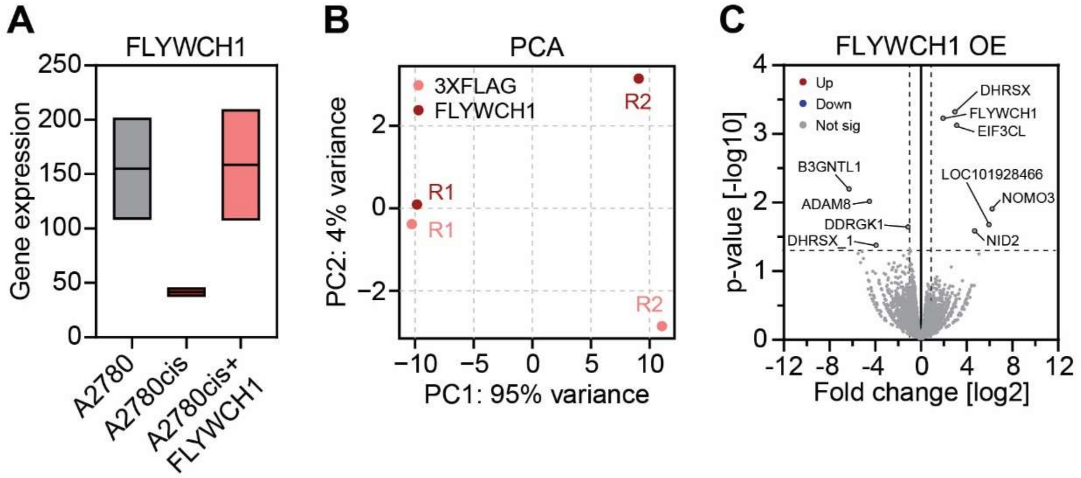

Figure S10. Overexpression of FLYWCH1 in resistant A2780cis cells has no significant effect on gene expression.
(A) Analysis of FLYWCH1 expression (normalised count) from RNA-seq data of sensitive A2780 cells compared to A2780cis cells with and without FLYWCH1 overexpression. Floating bars with min to max and line at mean, n=2. **(B)** Principle component analysis of RNA-seq data on A2780cis cells expressing FLYWCH1 or 3xFLAG as control. **(C)** Volcano plot showing gene expression changes in A2780cis cells following FLYWCH1 overexpression. Statistical significance was determined using DEseq2. Fold change in expression of plotted over the non-adjusted p-value to see mildly upregulated genes. Significantly up- or downregulation genes are indicated by colour based on the adjusted p-value.

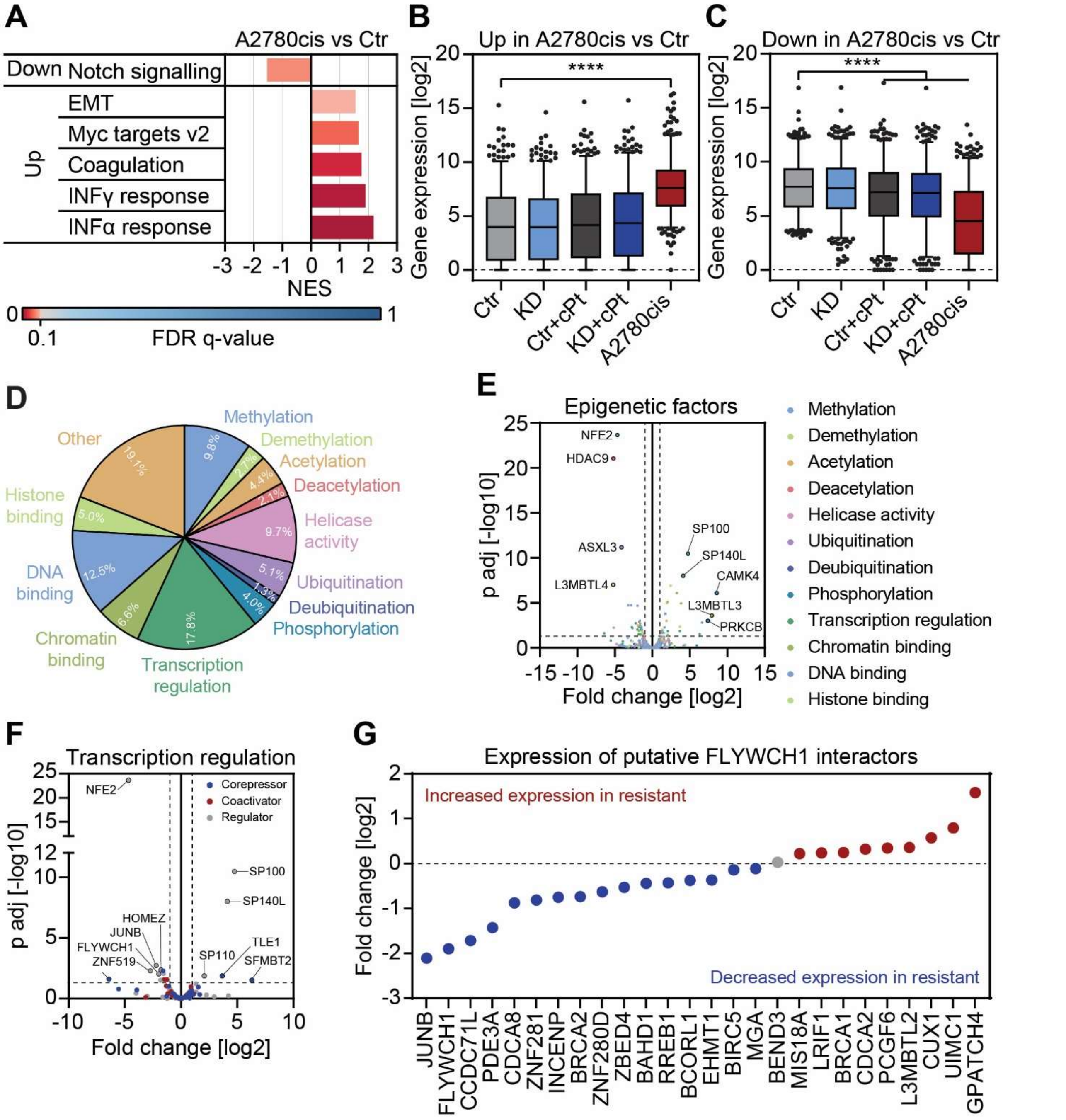

Figure S11. Resistance development is associated with changes in gene expression of epigenetic factors including putative FLYWCH1 interacting proteins.
(A) Gene-set-enrichment analysis for Hallmark pathways on significantly up- or downregulated genes in resistant A2780cis cells. All pathways with significant enrichment (FDR ≤ 0.1) are shown. **(B and C)** Average expression of all 787 upregulated (B) and 819 downregulated (C) genes in resistant A2780cis cells. Normalised count was averaged over the two independent shRNAs/repeats and plotted for each condition. Box plot with 2.5 and 97.5 percentiles, statistical analysis: Kruskal-Wallis test and Dunn's multiple comparisons test (****: p≤0.0001). **(D)** Categorization of epigenetic factors analysed in E based on their function. Genes were categorized based on their GO terms. **(E and F)** Volcano plot showing changes in gene expression between resistant A2780cis and sensitive A2780 cells. Genes were assigned functions based on their GO term as in D. E shows all genes associated with epigenetic regulation, F shows only genes associated with transcriptional regulation. Statistical significance was determined using DEseq2. **(G)** Expression change in putative FLYWCH1 interactors between sensitive and resistant cells. Putative FLYWCH1 regulators were extracted from previously published data (Santos-Barriopedro, van Mierlo, and Vermeulen 2021). Genes with decreased expression in A2780cis are marked in blue, genes with increased expression in red.

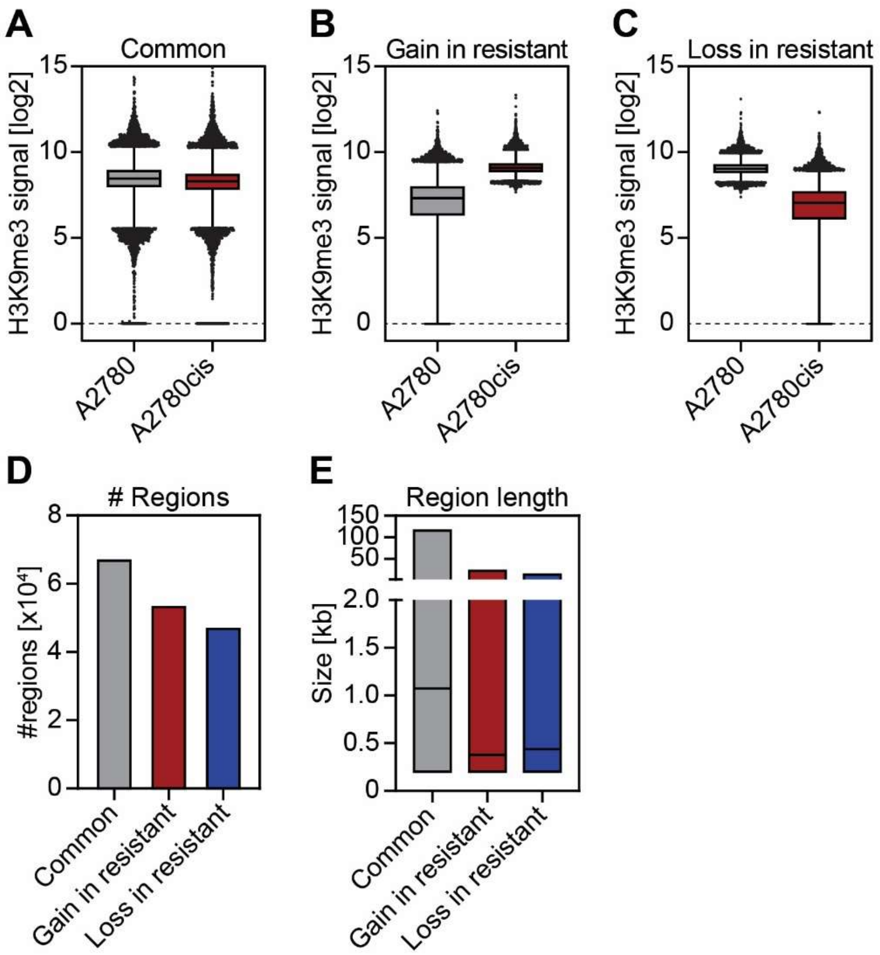

Figure S12. Resistant A2780cis cells show changes in H3K9m3 signal.
(A-C) H3K9me3 signal for all common and differential regions identified in Fig 5F. Average H3K9me3 signal per region was retrieved from bigwig files. Box plot with 1 and 99 percentiles. **(D)** Number of common and differential regions identified in Fig. 5F. **(E)** Size of the regions identified in Fig. 5F that are associated with common and differential H3K9me3 signal in resistant A2780cis cells. Floating bars with min to max and line at mean. Numbers of regions in each bar are displayed in D.

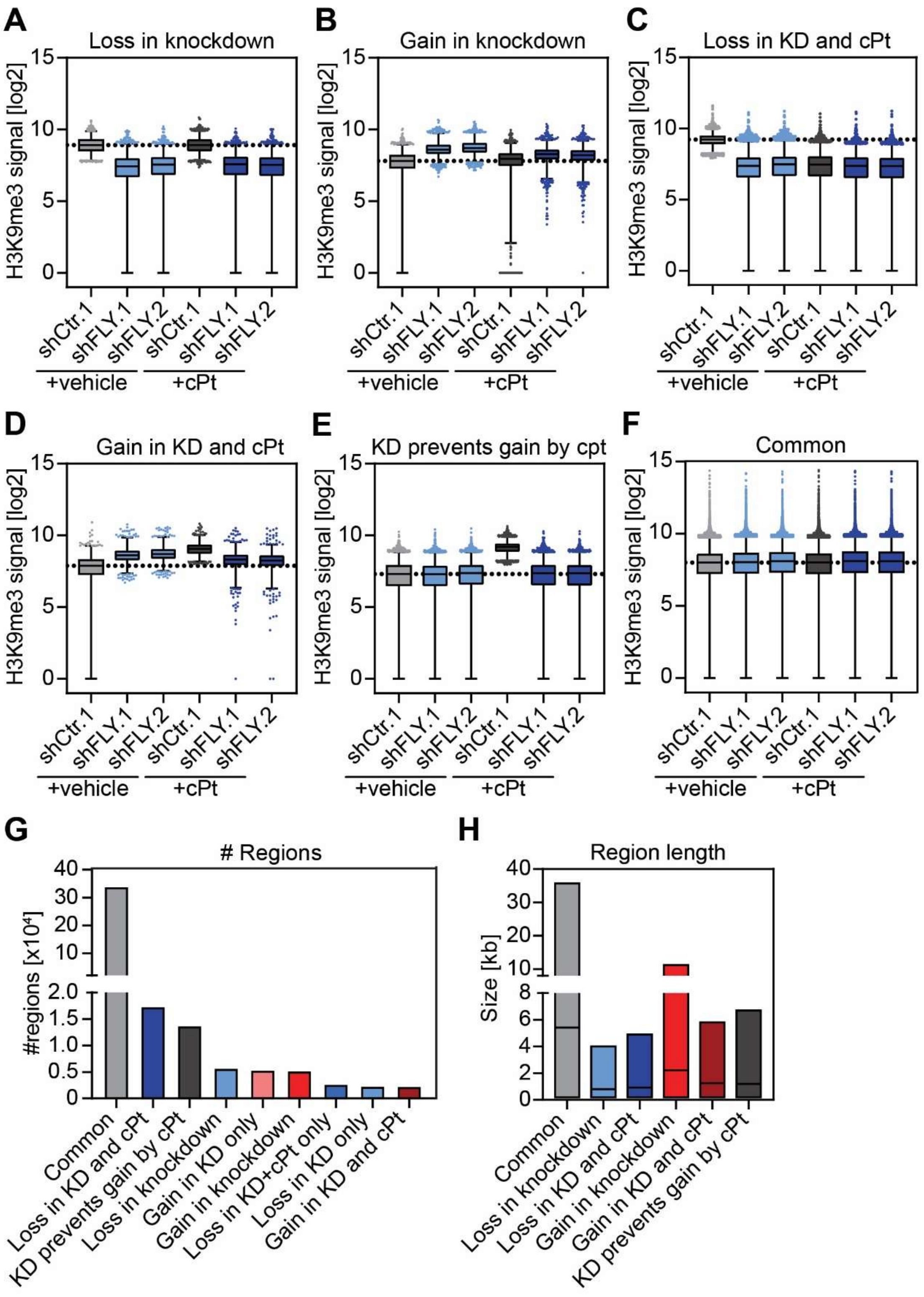

Figure S13. FLYWCH1 knockdown and early resistance development are associated with changes in H3K9me3.
(A-F) H3K9me3 signal for common and differential regions identified in Fig 6A. Average H3K9me3 signal per region was retrieved from bigwig files. Line in each graph indicates the median H3K9me3 signal for shCtr. Box plot with 1 and 99 percentiles. **(G)** Number of all common and differential regions identified in Fig. 6A. **(H)** Size of the regions identified in Fig. 6A that are associated with common and differential H3K9me3 signal in FLYWCH1 knockdown or early resistance development. Floating bars with min to max and line at mean. Numbers of regions in each bar are displayed in G.

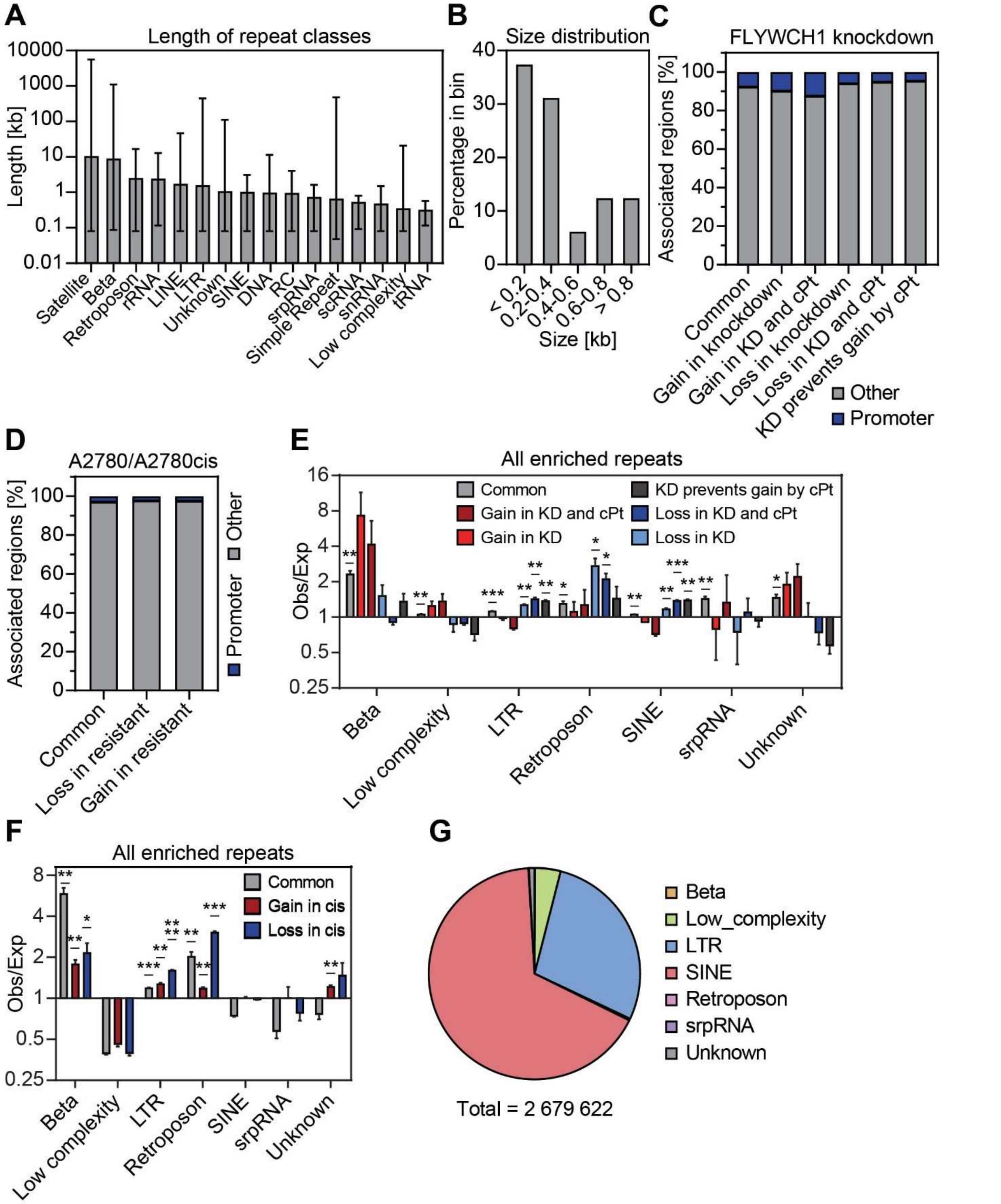

Figure S14. FLYWCH1 knockdown and resistance development lead to changes in H3K9me3 at repeat elements.

(A) Average length of repeat elements in all repeat classes. Bar graphs show mean with maximum and minimum values as error bars. **(B)** Distribution of average repeat size of repeat classes. Repeat classes were divided into bins based on their average repeat size. **(C and D)** Percentage of common and differential H3K9me3 peaks from FLYWCH1 knockdown and early resistance development (C) or A2780/A2780cis cells (D) overlapping with the area 1kb around gene promoters. **(E and F)** Observed/expected ratio of all common and differential regions from FLYWCH1 knockdown and resistance development (E) and A2780/A2780cis cells (F) with repeat elements of all repeat classes showing Obs/Exp ratio of at least one in one of the conditions in E. The observed incidence of the indicated repeat cluster was divided by the expected incidence if the peaks were distributed by random chance (shuffled bed file). Values higher than one indicate

that regions are associated with the respective repeat more often than expected by random chance, values less than one indicate a depletion of those regions from the repeat. Bed files were randomly shuffled three times for n=3 observed/expected ratios. Mean±SD, statistical analysis: one sample t-test was performed to test if the mean is larger than a hypothetical mean of one (*: $p \leq 0.05$, **: $p \leq 0.01$, ***: $p \leq 0.001$). **(G)** Distribution of repeat elements in the repeat classes analysed in E and F.

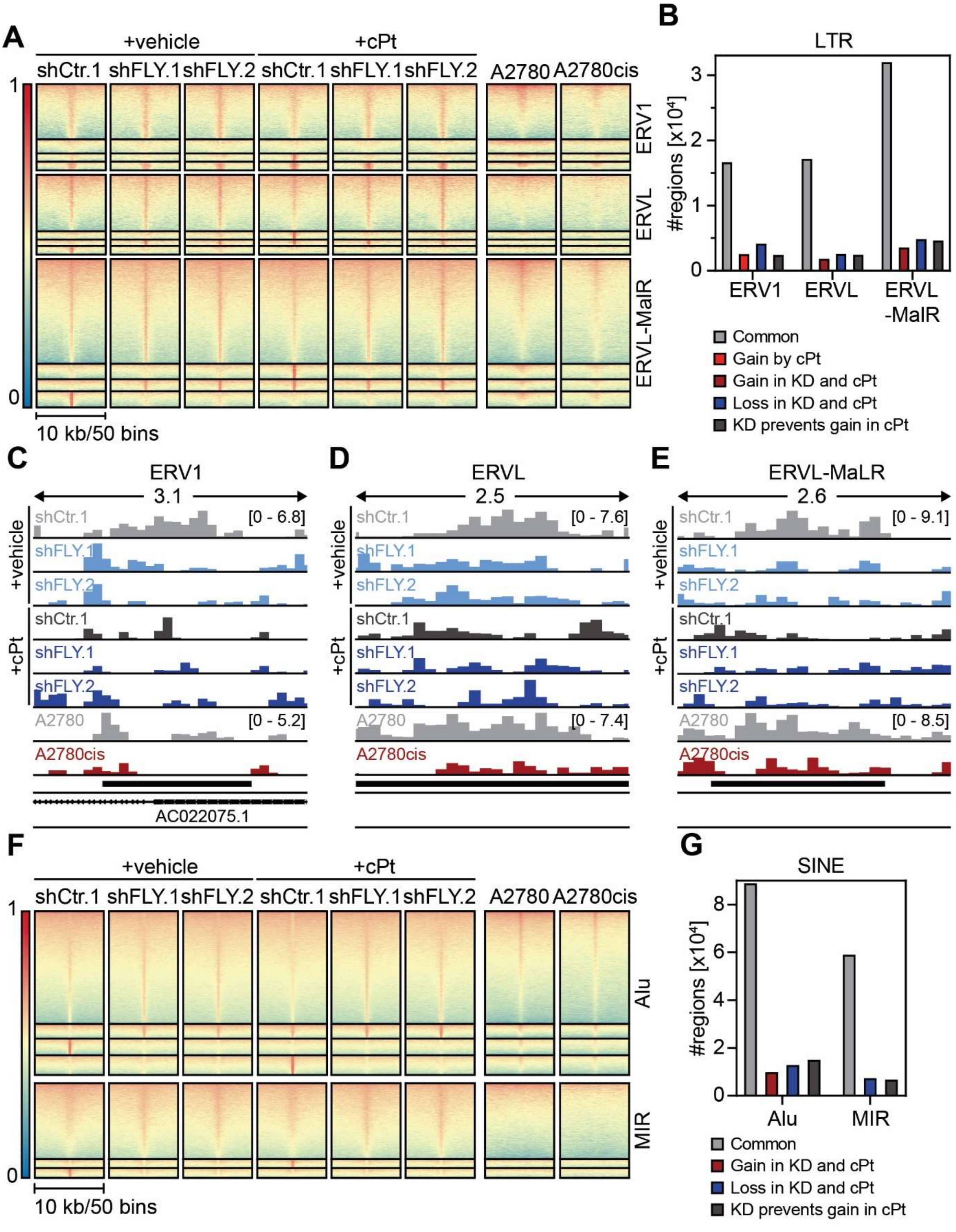

Figure S15. FLYWCH1 knockdown and resistance development are associated with changes at LTR and SINE elements.
(A and F) Heatmaps showing the average H3K9me3 ChIP-seq signal of control and FLYWCH1 knockdown cells, as well as cells that developed platinum-resistance dependent or independent of FLYWCH1 loss. ChIP-seq was performed on cells expression two independent hairpins (shCtr or shFLY) for 7 weeks and were treated with vehicle or 1 µM cPt for 6 weeks. Signals are sorted by the highest average signal in untreated shCtr cells and plotted with a 10 kb window in 50 bins around the peak centre. Regions with differential H3K9me3 signal in each repeat subfamily were identified separately by K-means clustering. A shows H3K9me3 ChIP-seq signal for the three biggest LTR subfamilies, F for the two biggest SINE subfamilies. **(B and G)** Number of regions with common and differential H3K9me3 signal identified in A and F. **(C-E)** Representative ChIP-seq tracks of H3K9me3 intensity for the data shown in A.

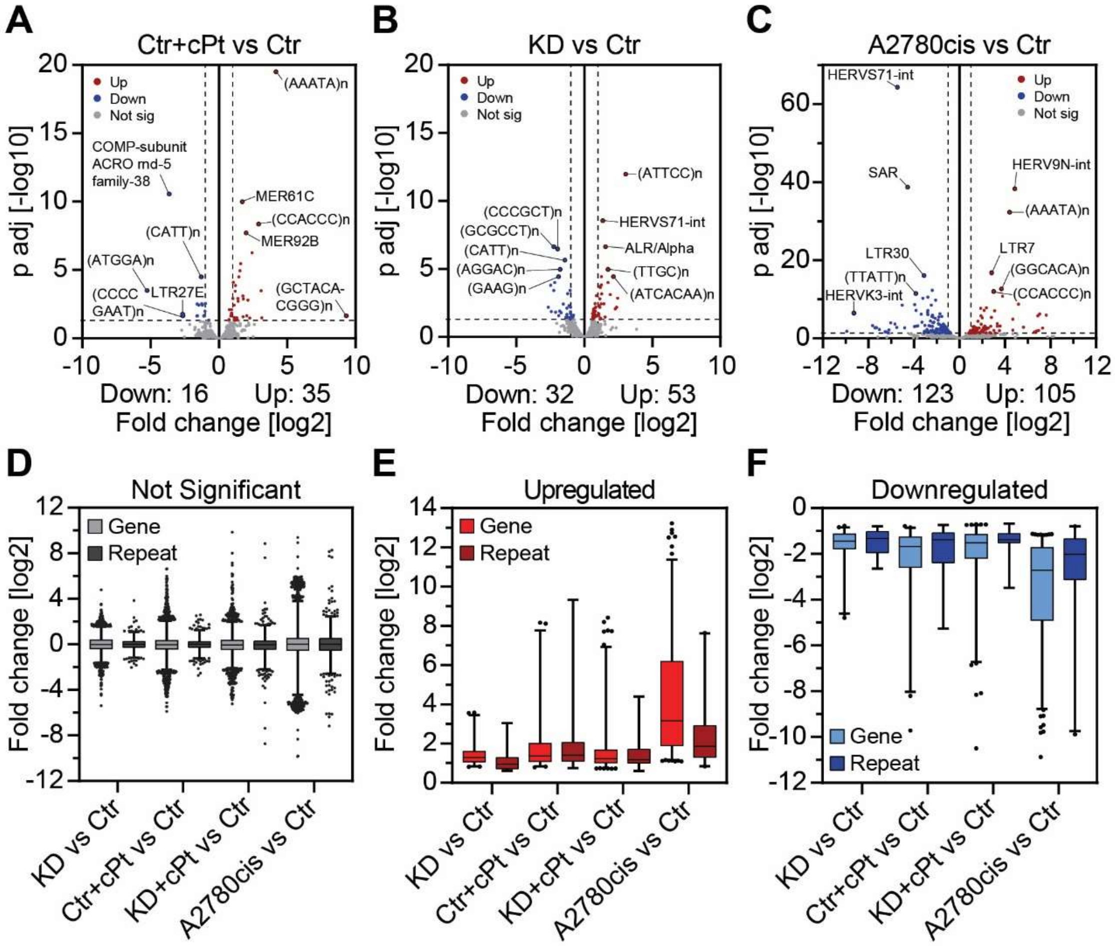

Figure S16. FLYWCH1 knockdown and resistance development leads to changes in repeat expression to a comparable level to the gene expression changes.
(A-C) Volcano plot showing changes in repeat expression in cells following early platinum-resistance development (A), FLYWCH1 knockdown (B) and long-term resistant A2780cis cells (C) relative to untreated control cells. Statistical significance was determined using DEseq2. Significantly up- or downregulated repeats are indicated by colour and their number is indicated below. **(D-F)** Fold change in expression of all genes and repeats without significant changes in expression (D), that are upregulated in expression (E) and that are downregulated in expression (F). Box plot with 1 and 99 percentiles.
